# Structural Isomerism and Enhanced Lipophilicity of Pyrithione Ligands of Organoruthenium(II) Complexes Increase Inhibition on AChE and BuChE

**DOI:** 10.3390/ijms21165628

**Published:** 2020-08-06

**Authors:** Jerneja Kladnik, Samuel Ristovski, Jakob Kljun, Andrea Defant, Ines Mancini, Kristina Sepčić, Iztok Turel

**Affiliations:** 1 Faculty of Chemistry and Chemical Technology, University of Ljubljana, 1000 Ljubljana, Slovenia; jerneja.kladnik@fkkt.uni-lj.si (J.K.); jakob.kljun@fkkt.uni-lj.si (J.K.); 2 Department of Biology, Biotechnical Faculty, University of Ljubljana, 1000 Ljubljana, Slovenia; samuel.ristovski@gmail.com; 3 Department of Physics, Bioorganic Chemistry Laboratory, University of Trento, 38123 Povo-Trento, Italy; andrea.defant@unitn.it (A.D.); ines.mancini@unitn.it (I.M.)

**Keywords:** Alzheimer’s disease, acetylcholinesterase, butyrylcholinesterase, cholinesterase inhibitor, molecular docking, organoruthenium(II) complexes, pyrithione derivatives

## Abstract

The increasing number of Alzheimer’s disease (AD) cases requires the development of new improved drug candidates, possessing the ability of more efficient treatment as well as less unwanted side effects. Cholinesterase enzymes are highly associated with the development of AD and thus represent important druggable targets. Therefore, we have synthesized eight organoruthenium(II) chlorido complexes **1a**–**h** with pyrithione-type ligands (pyrithione = 1-hydroxypyridine-2(1*H*)-thione, **a**), bearing either pyrithione **a**, its methyl (**b**-**e**) or bicyclic aromatic analogues (**f**–**h**) and tested them for their inhibition towards electric eel acetylcholinesterase (eeAChE) and horse serum butyrylcholinesterase (hsBuChE). The experimental results have shown that the novel complex **1g** with the ligand 1-hydroxyquinoline-2-(1*H*)-thione (**g**) improves the inhibition towards eeAChE (IC_50_ = 4.9 μM) and even more potently towards hsBuChE (IC_50_ = 0.2 μM) in comparison with the referenced **1a**. Moreover, computational studies on *Torpedo californica* AChE have supported the experimental outcomes for **1g**, possessing the lowest energy value among all tested complexes and have also predicted several interactions of **1g** with the target protein. Consequently, we have shown that the aromatic ring extension of the ligand **a**, though only at the appropriate position, is a viable strategy to enhance the activity against cholinesterases.

## 1. Introduction

Due to world’s aging population there is a progressive increase of the cases related to dementia [1]. The most common type of dementia that affects elderly population is Alzheimer’s disease (AD). AD is a neurodegenerative disorder, which is mainly associated with progressive disabling cognitive impairment such as memory loss and difficulties in speech; however, behavior and sleeping function are often impacted too, and changes in personality can also occur [2]. The symptoms described are the result of the neuropathologic changes, which have mainly been related to the elevated levels of amyloid-β (Aβ) plaques, neurofibrillary tangles of tau protein and neuronal death. However, in clinical trials the approach of diminishing Aβ plaque levels has been unsuccessful [3]. Still, the amyloid hypothesis cannot be discarded and should rather be complemented with other systems involved in the progress of this multifaceted disease. Therefore, the cholinergic hypothesis of AD was developed, which states that the cognitive decrease in patients with AD is also associated with the loss of cholinergic function in the central nervous system [4]. Normally, acetylcholine (ACh) as a neurotransmitter binds to acetylcholine receptors, which further triggers an intracellular cascade of events that maintains cognitive processes. AD patients face the decrease of these receptors, which is connected to the gradual loss of cholinergic neurons due to higher amount of Aβ plaques [5]. 

As mentioned, targeting Aβ plaques has not been successful. Therefore, the research has also focused on other targets—cholinesterases (ChEs)—which could more efficiently confront AD. In the cholinergic system, there are two ChEs, namely acetylcholinesterase (AChE) and butyrylcholinesterase (BuChE). Both ChEs have an ability to bind and cleave ACh so that neuron excitation can be, over time, cut off [6,7]. Both ChEs belong to serine hydrolase enzyme family with a catalytic triad in their active site consisted of Ser, His, and Glu. Although both ChEs exhibit a high degree of structural similarity, BuChE has a larger active pocket, allowing the accommodation of bulkier substrates, and affording bigger substrate specificity [8]. In the brain of healthy humans, AChE is the main enzyme responsible for the hydrolysis of ACh. During the AD progression, the activity of AChE remains the same or eventually declines, whereas the progressive increase of BuChE activity is observed. Therefore, the research and development of therapeutics with dual inhibition of both AChE and BuChE is urgently needed. The inhibition of both ChEs keeps the level of ACh high enough to maintain sufficient cholinergic transmission in the brain of AD patients and diminishes symptoms that can occur due to the lack of nicotinic acetylcholine receptors [7,9]. 

Currently, there are three ChE inhibitors approved by FDA for the treatment of AD. Two of them are donepezil and galantamine, which are selective non-competitive and competitive AChE inhibitors, respectively, and another one is rivastigmine that pseudo-irreversibly inhibits AChE as well as BuChE. All three compounds can penetrate through the blood brain barrier and inhibit the mentioned ChEs in the central nerve system [10,11]. Data from clinical trials have shown that these drugs stabilize or slow down cognitive and functional impairment; however, they only express modest overall benefits considering behavioral symptoms or clinical global change outcomes of AD patients. Moreover, ChE inhibitors were reported to cause serious adverse effects that are mainly connected to gastrointestinal problems, e.g. vomiting, nausea, diarrhea, and anorexia [12]. Therefore, it is of great importance to develop new improved drug candidates with better activity against ChE in the central nervous system and less unwanted effects on the peripheral nervous system [13].

**Scheme 1:**
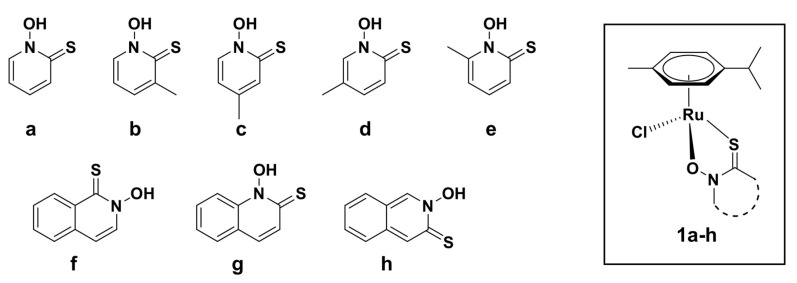
Pyrithione (**a**), its methyl (**b**–**e**), and bicyclic aromatic analogues (**f**–**h**) and their corresponding organoruthenium(II) chlorido complexes (**1a**–**h**).

Metals also play important role in the development of AD. Abnormal elevated concentrations of iron, copper, and zinc were found in amyloid plaques in post-mortem brain tissues of the individuals with AD [14]. Moreover, it is also frequently mentioned that aluminum is somehow connected to AD, though its role is still not clearly known [15]. Therefore, drug design of chelators to restore the homeostasis of mentioned metals was also considered as one of the possible therapeutic approaches in treating AD [14]. However, as mentioned, cholinergic system seems to be the most promising therapeutic field to combat AD. Some ruthenium complexes, mainly ruthenium(II) polypyridyl compounds, have also been examined as AChE inhibitors [16,17,18], and some of them additionally inhibit Aβ aggregation [19,20,21]. Recently, we have reported a potent chlorido organoruthenium(II) complex with pyrithione **1a** (Scheme 1) (**a**—pyrithione, also 1-hydroxypyridine-2(1*H*)-thione or 2-mercaptopyridine *N*-oxide) [22], which is an excellent reversible competitive inhibitor of three ChEs, namely electric eel AChE (eeAChE), human AChE (hAChE), and horse serum BuChE (hsBuChE) with low micromolar IC_50_ values (5.01 µM, 25.06 µM, and 7.52 µM, respectively). Complex **1a** also inhibited enzyme glutathione-S-transferase, which is involved in chemotherapeutic drug resistance. Moreover, complex **1a** showed no cytotoxicity against non-cancer HUVEC and NHEK-1 cells and also did not express any unwanted physiological response on the neuromuscular transition at pharmaceutically relevant concentrations. The latter finding is of the particular importance, as many AChE inhibitors show many adverse effects, including uncontrolled muscle contraction and the failure of neuromuscular transmission in the peripheric nervous system [23]. Another important aspect when developing new potential drugs is the behavior of the compound in human serum, which has also been thoroughly investigated for the complex **1a**. Our data have shown that complex **1a** mainly interacts with human serum albumin (70%) and, though more weakly, also with immunoglobulins G (5%), whereas 25% of the compound **1a** remains unbound [24].

Taking into consideration all mentioned positive outcomes of organoruthenium(II)-pyrithionato systems, we have decided to further elucidate the potential of such types of complexes on the ChE enzymes. There have been several reports of AChE inhibitors that take part not only in affecting ChE activity, but also affect non-neuronal functions such as the regulation of cell proliferation, differentiation, and apoptosis. Clinically used drugs for the treatment of AD, e.g., galantamine and donepezil, have also been tested for such effects [25], where galantamine has induced antiproliferative activity against 3T3 cells and donepezil has triggered apoptosis on HL-60 human promyelocytic leukemia cells [26]. Moreover, there are also some chemotherapeutics, e.g., irinotecan [27], cyclophosphamide [28], sunitimib [29], and even cisplatin [30], that have expressed potent AChE inhibition. Compounds with such dual anticholinesterase as well as anticancer activity can be taken into consideration for the treatment of AD and cancer, concomitantly. Additionally, there is an interesting correlation between AD and cancer occurrence, as AD patients suffer less from cancer and cancer patients less from AD development. Such statistics points to some similar mechanisms of action involved in both disease processes, which can be used for the development of new efficient drugs for these two indications [31]. Apart from previously synthesized complexes **1a**–**e**, which have been evaluated for their anticancer properties [32], we have extended our synthesis to bicyclic aromatic pyrithione analogues **f**–**h** and prepared their new chlorido organoruthenium(II) complexes **1f**–**h** (Scheme 1). In the present work, novel experimental results on the inhibition towards eeAChE and hsBuChE by the compounds **a**–**h** and **1a**–**h** are shown, supported by a docking study of the complexes **1a**–**h** on *Torpedo californica* AChE (TcAChE). Molecular docking is indeed a useful tool used in drug discovery, very often able to relate to the experimental biological evaluation. It gives an indication on the relative energy values of the complexes between a macromolecular target and each ligand of a series of molecules, as well as on the number and types of interactions involved in the receptor site. To our knowledge, this is the first expanded study of the organoruthenium(II) pyrithione complexes on ChEs with an aim to provide comprehensive results that are needed in the development of new improved drugs for AD in the field of organometallic compounds.

## 2. Results and Discussion

### 2.1. Synthesis and Crystal Structures

Ligand **a** is a commercially available compound, whereas the ligands **b**–**e** and their corresponding chlorido complexes **1a**–**e** were prepared according to Kladnik et al. [32]. Ligands **f**–**h** were prepared following the protocol reported by Cohen et al. [33,34] with modifications described in Section 3.3. New complexes with (iso)quinoline-derived pyrithione ligands **1f**–**h** were synthesized by reacting the ruthenium cymene precursor (RuCym, [Ru(*p*-cymene)Cl_2_]_2_) with the appropriate pyrithione analog (**f**–**h**) in the presence of the base NaOMe. The reaction mixtures were stirred overnight in dichloromethane (DCM). After the reaction was completed (monitored by TLC) the solvent was evaporated and the reaction mixture was purified by column chromatography to eliminate all impurities, including the precipitated byproduct NaCl. After combining all fractions containing the product, the mobile phase was evaporated and the oily residue redissolved in DCM and evaporated again to completely remove methanol from mobile phase and facilitate precipitation. Complexes **1f**–**h** precipitated from DCM/*n*-heptane mixture and the red precipitates were filtered under reduced pressure, followed by drying at 45 °C overnight. The complexes are air, moisture, and light stable. 

**Figure 1:**
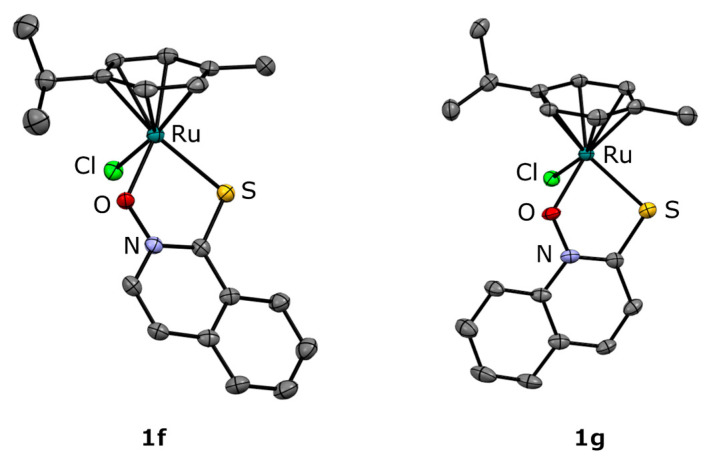
Crystal structures of the complexes **1f** and **1g**. The thermal ellipsoids are drawn at 35% probability level. The hydrogen atoms are omitted for better clarity of presentation.

All compounds were characterized by ^1^H NMR, UV-Vis, and IR spectroscopy as well as high-resolution electrospray ionization mass spectrometry (ESI-HRMS) and elemental analysis (C, H, N). New crystal structures for **1f** and **1g** were obtained by X-ray diffraction on monocrystals (Figure 1, Table S1, Figure S1).

The crystal structures of **1f** and **1g** show the typical piano-stool geometry of organoruthenium-cymene complexes. The ligands are expectedly coordinated through the oxygen and sulfur atoms of the thiohydroxamate moiety with bond lengths and values comparable to those of analog complexes of methyl-substituted pyrithiones. Interestingly, in the case of compound **1g** the crystal packing of the compounds into layers is stabilized mainly by π-stacking interactions (Figure S2), while compound **1f** exhibits weak hydrogen bonds between the cymene hydrogens and the coordinated oxygen atoms also observed in the parent ruthenium-pyrithione complex.

### 2.2. Stability of Organoruthenium(II) Pyrithione Complexes

The isolated chlorido complexes **1a**–**h** are neutral molecules. However, when considering metal complexes as potential therapeutics the investigation of their stability in aqueous media is of utmost importance. It is well-known that organoruthenium(II) complexes bearing monodentate halide ions (e.g., Cl^-^) act as prodrugs in biological media, as halides are prone to the substitution with other surrounding solvent molecules [35,36,37,38]. These processes are often complex and best described as equilibria of more metal species in the final solutions. As reported by many other researchers, dealing with similar organometallic systems, Ru–Cl is a labile bond, subjected to give fast production of Ru–OH_2_ or Ru–OH species in aqueous media. As water molecules are neutral entities, newly formed Ru-OH_2_ species are positively charged, which might ease the electrostatic interactions between metal complexes and target proteins [35]. Moreover, when performing biological assays, solubilizing agents such as DMSO or ethanol are often added to prepare stock solutions. These molecules can, however, also react with metallodrugs possessing labile ligands [39]. In our previous studies, we have found that the behavior of the complexes depends on the type of the ligand and together with all above mentioned factors affect the kinetics of interactions with biomolecules, and thus, also their biological activity [40,41,42]. Previously, we have already reported stability investigations of some organoruthenium(II)-pyrithionato complexes [22,24,32] as well as of structurally related organoruthenium(II) compounds [40,41,42] by NMR, MS, and UV-Vis spectroscopies. Firstly, we have shown by ^1^H NMR that compound **1a** is stable in DMSO-d_6_/D_2_O solvent system [22] and later also confirmed the stability of **1a** complex in the D_2_O solution containing NaCl [24]. Furthermore, in another study on organoruthenium(II)-pyrithionato systems, we reported similar NMR experiments for the compound **1b**, and in both cases the negligible release of *p*-cymene was observed. Moreover, none of the experiments showed the presence of the free *O,S*-ligand, which indicated an exceptional stability of the formed adducts. With the addition of NaCl to the investigated solutions high concentration of the halide ion in the extracellular fluids was mimicked, which may suppress mentioned hydrolysis. Therefore, it is speculated that the activation of the complexes to positively charged species occurs inside the cell with lower NaCl concentrations. The stability of **1b** was further supported by the UV-Vis investigation in aqueous media where compound **1b** resulted stable in phosphate buffer solution, cultural media RPMI and FP-RPMI, as well as plasma [32].

For the complexes **1e** and **1g** we have first checked whether the compounds remain stable under the conditions used for enzymatic assay on ChEs, where compounds are first dissolved in 100% EtOH and then progressively diluted with potassium phosphate buffer (*v/v* % of EtOH between 0.02–6.25%, where the small amount of added EtOH does not influence enzyme activity). All assays were carried out immediately after the solution preparation. Therefore, we have prepared 6.25% EtOH-d_6_/D_2_O solutions containing 80.2 mM K_2_HPO_4_ and 19.8 mM KH_2_PO_4_ (600 µL) for each complex (circa 4 mg) and recorded ^1^H NMR spectra immediately after the preparation of the solution and later after one hour. From the Figure S3, we can see that tested methyl as well as bicyclic aromatic pyrithione complex remain stable under conditions used. Thus, we can conclude that the tested complexes are responsible for enzymatic inhibition. Furthermore, we have also followed the stability of the complex **1e** and **1g** in D_2_O, containing 140 mM NaCl to assess the influence of high chlorido concentration present in plasma. After one day we could observe negligible changes in the spectra in case of both complexes (Figures S4B and S5B). The same was observed for the stability of the complexes, recorded only in D_2_O without NaCl present (Figures S4A and S5A), suggesting that under extra- and intracellular conditions the complexes should remain intact.

**Figure 2:**
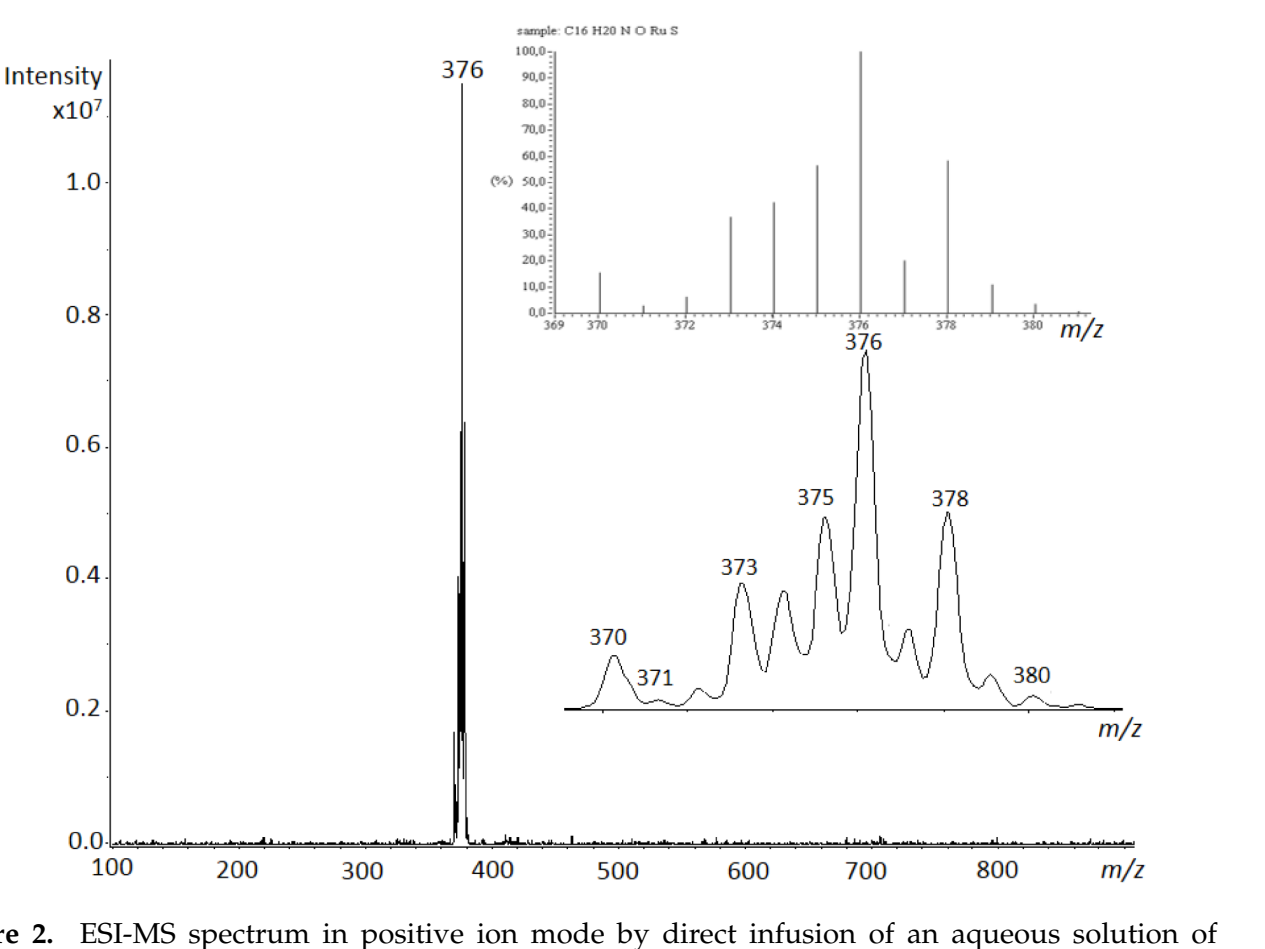
ESI-MS spectrum in positive ion mode by direct infusion of an aqueous solution of compound **1b**, with the isotopic cluster corresponding to [M − Cl]^+^ ion highlighted and compared with the simulated cluster for C_16_H_20_NORuS composition.

An additional evidence was provided by electrospray ionization-mass spectrometry (ESI-MS), taking advantage by sensitivity and soft ionization method of this technique, which allows to reveal non-covalent interactions with an efficient detection across a range of solvents, which we have already reported in some similar systems for organoruthenium(II) compounds [41,42]. In detail, ESI-MS analysis was carried out in positive ion mode by direct infusion from an aqueous solution of **1b** prepared immediately before measurement and after three days. The same spectrum was observed showing only the isotopic pattern for the [M − Cl]^+^ ion (Figure 2), which is in good agreement with the ^1^H NMR stability data obtained immediately and after three days for the complexes **1e** and **1g** in D_2_O, where the complexes remain the main present species (Figures S4A and S5A). Replacing water with a MeOH solution of **1b** gave identical behavior. Moreover, similar analyses changing solvent and time displayed only [M − Cl]^+^ ion with clusters at *m*/*z* 376 for the isomeric compounds **1c**–**e**, at *m*/*z* 362 for **1a** and at *m*/*z* 412 for **1f–h**.

### 2.3. Enzymatic Assays

We have already reported that compound **1a** exerts very good ChE-inhibitory activity [23]. Therefore, an expanded library of organoruthenium(II)-pyrithione complexes was prepared to get more extensive insight into their further potential in the treatment of AD. The obtained compounds **a**–**h** and **1a**–**h** were first investigated for their inhibition against eeAChE and hsBuChE. By introducing the methyl group, we wanted to analyze the potential influence of small structural differences on the ChE-inhibitory activity. In fact, some methyl-substituted pyrithione ligands as well as their organoruthenium(II) complexes were already found to exert stronger biological activities than their non-methylated analogue, as reflected for instance in the inhibition of carbonic anhydrase [33] or in anticancer effects [32], respectively.

We have also decided to prepare pyrithione analogues with extended aromatic rings (**f**–**h**, **1f**–**h**) to increase lipophilicity of the compounds, which is an important factor as optimal lipophilicity enhances chances in the success of drug discovery [43]. Therefore, (iso)quinoline-derived pyrithione ligands **f**–**h** were resynthesized and their novel organorutehinium(II) complexes were prepared to check whether this strategy could also be applied in the cholinergic system. 

The IC_50_ values and inhibition constants (*K_i_*) of the ligands **a**–**h** and organoruthenium(II) chlorido complexes **1a**–**h** against eeAChE and hsBuChE are presented in Table 1. Ligands **a**–**g** were not active against eeAChE or hsBuChE and ligand **h** was only poorly active against eeAChE and hsBuChE, whereas complexes **1a**–**h** showed a reversible competitive type of inhibition of both tested enzymes, with inhibitory constants in the low micromolar range. Comparing IC_50_ values of our compounds with those obtained with neostigmine bromide, which is a golden standard in ChE inhibition studies, it can be observed that complex **1g** has comparable activity against eeAChE, whereas tested complexes **1a**–**h** resulted as more potent inhibitors of hsBuChE.

Complexes inhibit eeAChE in low micromolar range with the lowest and the highest IC_50_ values of 4.9 µM and 14.3 µM for **1g** and **1h**, respectively. The introduction of electron-donating methyl group at *meta* and *ortho* positions of the *N*-oxide group of the complexes **1d** and **1e** resulted in the slight increase of the inhibitory potential towards eeAChEs in comparison to **1a**. Considering hsBuChE inhibition, parallels can be drawn between investigated ChEs. Generally, the highest inhibition degree against hsBuChE was obtained for the complex **1g** (IC_50_ = 0.2 µM), and among methyl substituted complexes also with **1e** (IC_50_ = 0.7 µM). The lowest inhibition against hsBuChE was achieved with complex **1c**, which, however, still remains in the low micromolar range with an IC_50_ value of 2.7 µM. Generally, the IC_50_ values obtained with hsBuChE are lower than those obtained with eeAChE. In particular, all three complexes bearing bicyclic aromatic ligands **f–h** exert their BuChE-inhibitory activities at very low concentrations. The reason behind might be the difference in the active gorge size of the eeAChE and hsBuChE, which is larger in case of the latter. Therefore, bigger complexes have easier access to the catalytic triad and more space to adjust the most convenient position and interactions with other amino acid residues to fit the hsBuChE active gorge.

Importantly, the results above point to some preliminary structure activity relationship. In both cases, methyl-substituted complexes **1d** and **1e** are better inhibitors of eeAChE as well as hsBuChE in comparison to unsubstituted **1a** complex. Furthermore, when those two methyl groups at *ortho* and *meta* position of the *N*-oxide group are connected via the benzene ring, thus forming complex **1g**, the inhibition against eeAChE and hsBuChE increases even more. Therefore, the position of the fused benzene ring regarding to the thiohydroxamic group on pyrithione **a** importantly influences inhibition, especially when taking into consideration the inhibition of eeAChE, where complex **1g** exerts the highest, whereas **1h** the lowest inhibition among all tested complexes. Thus, not only the increased lipophilicity, but also the structural isomerism plays a very important role.

### 2.4. Computational Study

Docking calculations were carried out to support experimental data on AChE inhibition, while the lack of results on the tridimensional structure for hsBuChE prevented such computational approach. In order to understand the interactions of the compounds **1a**–**h** with the active sites of AChE, docking calculations were carried out using the AutoDock program. eeAChE was used in the experimental study, but the corresponding X-ray structure was available at a very low resolution (PDB, ID: 1C2O, 1C2B, and 1EEA, at 4.2–4.5 Å), though only as native enzymes without ligand. To overcome this problem, TcAChE 6G1V at 1.8 Å resolution could be used as a model system [44], relying on the fact that a comparison between these two structures showed a 0.59 TM-score indicating approximately the same fold [45]. Above all, the two enzymes showed the same amino acid residues in the active sites, except for tyrosine 330 in eeAChE, replaced by phenylalanyl 330 in TcAChE, indicating very similar receptor ability (Figure S18). 

Table 2 reports the data by docking calculation for compounds **1a**–**h** and neostigmine bromide used as the reference in experimental assays. Note that in the case of unusual elements, such as some transition metals, the calculation by AutoDock needs parameters in addition to the standard approach. Therefore, in this study on organoruthenium(II) complexes, the results must be taken with due caution. Despite this, we have obtained a good correlation between experimental data (with IC_50_ values in the range 5–14 µM and 4.3 µM for neostigmine bromide) and the docking results including energy values (from −10 to −7 Kcal/mol, and −7.0 Kcal/mol for neostigmine bromide), and both the number and type of interactions.

**Figure 3:**
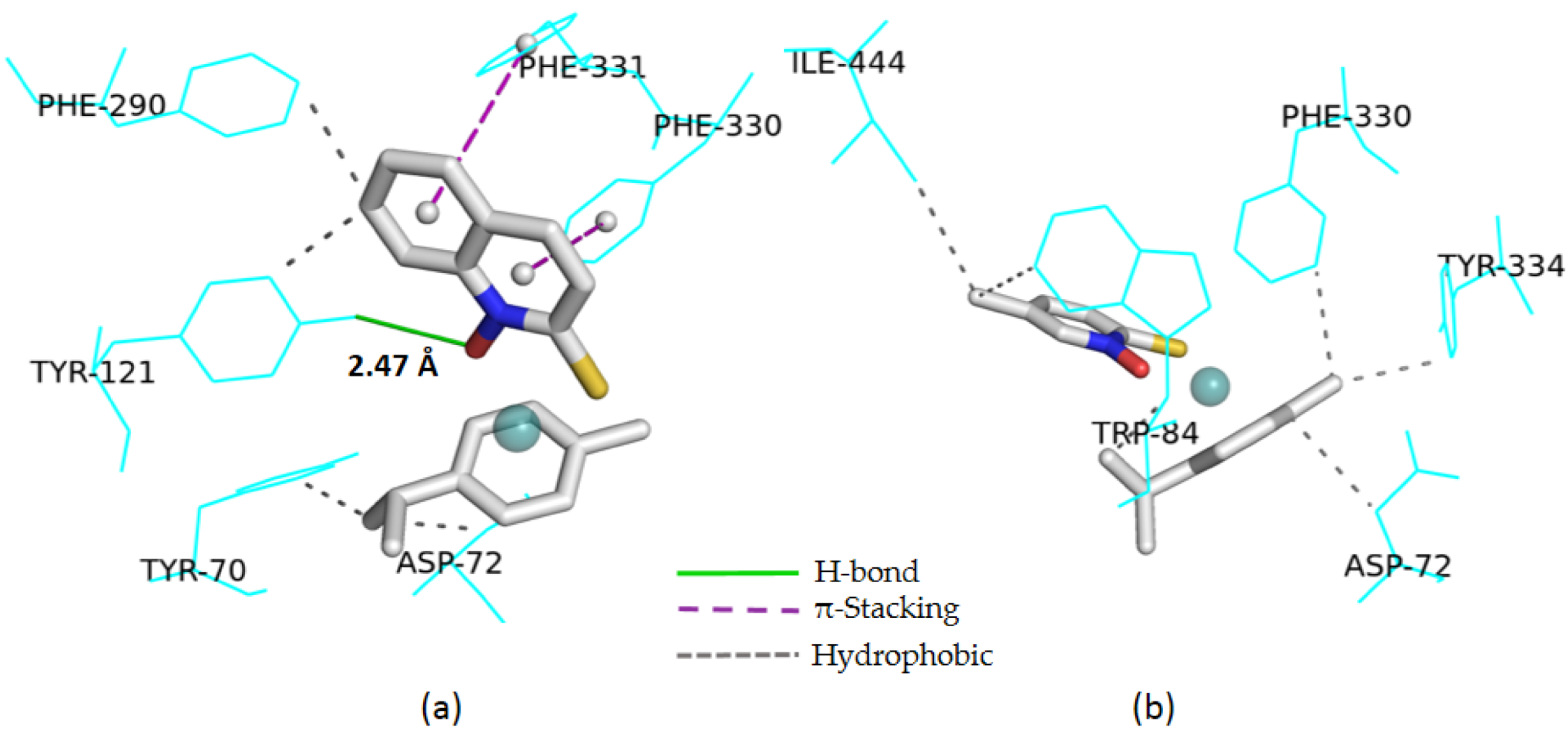
Three-dimensional views of the interactions for the compounds **1g** (**a**) and **1d** (**b**) with TcAChE by docking calculations with indications of all interactions.

In detail, the best AChE inhibition observed for the complex **1g** with 1-hydroxyquinoline-2-(1*H*)-thione ligand is in line with the lowest calculated energy value, combined with the presence of a H-bond involving tyrosine 121 even at a shorter distance than the one observed for neostigmine bromide, two **π-**stackings, and a series of hydrophobic interactions. In the methyl-pyrithione series, compound **1d** displayed a good enzyme inhibition, associated to favorable docking results, including a low energy value and a high number of hydrophobic interactions (Figure 3).

Based on the most promising data obtained for **1g** and **1d**, the key descriptors to evaluate their drug-likeness have been regarded by ADME prediction (Figure S19). Both compounds show a good gastrointestinal absorption, which is essential for an oral drug administration. Moreover, for complex **1d** it was also shown to penetrate the blood-brain barrier, a requirement for therapeutics active on the central nervous system. 

## 3. Materials and Methods

### 3.1. Chemicals

Starting materials for the syntheses of the ligands **a**–**h** and other reagents for the synthesis of the complexes **1a**–**h**, as well as the solvents, were purchased from commercial suppliers (Fluorochem, Hadfield, UK; Strem Chemicals, Cambridge, UK; Merck, Darmstadt, Germany) and used as received, with the exception of CHCl_3_ used in the reactions of *N*-oxidation for **f′**–**h′**, which was dried over Na_2_SO_4_ before use. For monitoring the progress of the reactions pre-coated TLC sheets ALUGRAM^®^ SIL G/UV_254_ (Macherey-Nagel, Düren, Germany) were used and visualized under UV light. Merck Silica gel 60 (35–70 µm) as a stationary phase was employed to perform column chromatography.

### 3.2. Characterization

The physicochemical characterization of prepared compounds was performed by ^1^H NMR spectroscopy, CHN elemental analysis, high resolution electrospray ionization mass spectrometry (ESI-HRMS), infrared (IR), UV–Vis spectroscopy, and X-ray diffraction on monocrystal. ^1^H NMR spectra for **f′**–**h′**, **f**–**h**, and **1f**–**h** together with IR spectra for **1f**–**h** can be found in the Supplementary Materials (Figures S6–S14 and S15–S17, respectively). Characterization data for the compounds **a**–**e** and **1a**–**e** was previously reported by Kladnik et al. [32].

^1^H NMR spectra were recorded using Bruker Avance III 500 spectrometer at room temperature at 500 MHz. Chemical shifts of ^1^H NMR spectra are referenced to deuterated solvent residual peaks of CDCl_3_, DMSO-d_6_, and D_2_O at 7.26, 2.50, and 4.79 ppm, respectively. The splitting of the proton resonances is defined as s = singlet, d = doublet, t = triplet, q = quartet, hept = heptet, m = multiplet, and bs = broad signal. Chemical shift (*δ*) are given in ppm and coupling constants (*J*) in Hz. NMR data processing was carried out with MestReNova version 11.0.4. Infrared spectra were recorded with a Bruker FTIR Alpha Platinum ATR spectrometer and high-resolution mass spectra (HRMS) on an Agilent 6224 Accurate Mass TOF LC/MS instrument. Elemental analyses were performed on a PerkinElmer 2400 II instrument (C, H, N). UV-Vis spectra for compounds were obtained on PerkinElmer LAMBDA 750 UV/Vis/near-IR spectrophotometer. Electrospray ionization (ESI)-MS mass spectra by direct infusion of aqueous or methanol solution of the compounds **1a**–**h** were recorded using a Bruker Esquire-LC spectrometer (drying gas N_2_, 4 L/min, scan range *m/z* 100–1000, Skim1 15.2 V). Isotopic clusters were simulated by home-made software (University of Trento, Italy).

X-ray diffraction data was collected on an Oxford Diffraction SuperNova diffractometer with Mo/Cu microfocus X-ray source (Kα radiation, λ_Mo_ = 0.71073 Å, λ_Cu_ = 1.54184 Å) with mirror optics and an Atlas detector at 150(2) K. The structures were solved in Olex^2^ graphical user interface [46] by direct methods implemented in SHELXT and refined by a full-matrix least-squares procedure based on F^2^ using SHELXL [47]. All non-hydrogen atoms were refined anisotropically. The hydrogen atoms were placed at calculated positions and treated using appropriate riding models. The crystal structures have been submitted to the CCDC and have been allocated the deposition numbers 2006351 and 2006352 for the compounds **1f** and **1g**, respectively.

### 3.3. Syntheses

Ligands **f–h** were prepared according to the known procedure [33] with some modifications described below. General schemes for the synthesis of the ligands **f**–**h** and complexes **1f**–**h** are presented in the Supplementary Materials (Scheme S1 and S2). Chlorido complexes **1f**–**h** were prepared according to the adjusted previously reported procedures from our group [32]. 

General procedure **f′**–**h′** (*N*-oxidation). Precursors 1-bromoisoquinoline, 2-bromoquinoline or 3-bromoisoquinoline (400 mg, 1 mol. equiv.) were combined with *m*-chloroperoxybenzoic acid (948 mg, *m*-CPBA, 2 mol. equiv., 70% purity) in CHCl_3_ (10 mL) and stirred at room temperature for 5 hours and overnight for **f′** and **g′**–**h′**, respectively. The solution was first washed with 1 M NaOH (1 × 50 mL), and then, the water phase was extracted with CHCl_3_ (2 × 25 mL). The combined organic phases were dried over sodium sulfate, filtered, and evaporated under reduced pressure. The residue was purified by column chromatography on silica gel, eluting with 2% MeOH/DCM or 5% MeOH/DCM for **f’**–**g’** or **h’**, respectively. After the solvent removal, a white solid was obtained that was dried at 45 °C overnight. 

*1-Bromoisoquinoline N-oxide* (**f’**). Yield: 59%. ^1^H NMR (500 MHz, DMSO-d_6_): *δ =* 8.39 (d, 1H, *J* = 7.1 Hz, Ar–*H*), 8.02 (t, 2H, *J* = 7.5 Hz, Ar–*H*), 7.98 (d, 1H, *J* = 7.1 Hz, Ar–*H*), 7.83–7.78 (m, 1H, Ar–*H*), 7.72–7.66 (m, 1H, Ar–*H*).

*2-Bromoquinoline N-oxide* (**g’**). Yield: 19%. ^1^H NMR (500 MHz, DMSO-d_6_): *δ =* 8.55 (d, 1H, *J* = 8.8 Hz, Ar–*H*), 8.12 (d, 1H, *J* = 7.7 Hz, Ar–*H*), 7.94–7.84 (m, 3H, Ar–*H*), 7.80–7.75 (m, 1H, Ar–*H*).

*3-Bromoisoquinoline N-oxide* (**h’**). Yield: 85%. ^1^H NMR (500 MHz, DMSO-d_6_): *δ =* 9.23 (s, 1H, Ar–*H*), 8.57 (s, 1H, Ar–*H*), 7.90 (t, 2H, *J* = 8.4 Hz, Ar–*H*), 7.70–7.62 (m, 2H, Ar–*H*).

General procedure for **f**–**h** (thiolation). To appropriate bromo(iso)quinoline *N*-oxide **f’**–**h’** (200 mg) saturated NaSH_(aq)_ (10 mL) and water (10 mL) were added and left to stir at room temperature overnight for **f**–**g** or on reflux for 6 hours for **h**. The solution was acidified with 4 M HCl_(aq)_ to pH ~1, followed by the immediate extraction with CHCl_3_ (2 × 30 mL). The combined organic layers were dried over sodium sulphate, filtered, and evaporated. The residue was triturated with acetone and the byproduct elemental sulphur S_8_ was filtered off. After evaporation of the mother liquor solution, a yellow-greyish solid was obtained. Ligands without further purification were used for the complexation with ruthenium precursor RuCym. In case the ligands were needed for the biological assays, column chromatography on silica gel was performed, eluting with hexane/ethyl acetate = 9/1 or 9.5/0.5 for **f** or **g**, respectively, to afford a yellow-greyish solid. Yields given below are from the obtained compounds before their purification by column chromatography. Yields for **f**–**g** after purification varied between 40–70% as obtained thiones are sensitive to silica gel and partly decompose, which was similarly observed in a previous study [48]. For the ligand **h,** we were not able to obtain analytically pure compound, as it totally degrades on silica gel; however, Adamek et al. reported the same compound **h** could be obtained with 4% yield using a reverse chromatography and crystallization [34].

*2-Hydroxyisoquinoline-1-(2H)-thione* (**f**). Yield: 88%. ^1^H NMR (500 MHz, DMSO-d_6_): *δ =* 12.10 (s, 1H, N–O*H*), 8.72 (d, 1H, *J* = 8.3 Hz, Ar–*H*), 8.23 (d, 1H, *J* = 7.3 Hz, Ar–*H*), 7.90 (d, 1H, *J* = 7.9 Hz, Ar–*H*), 7.83–7.78 (m, 1H, Ar–*H*), 7.73–7.68 (m, 1H, Ar–*H*), 7.31 (d, 1H, *J* = 7.3 Hz, Ar–*H*). ESI-HRMS (CH_3_CN) *m/z* for [M + H]^+^ (found (calcd)): 178.0329 (178.0321). Anal. Calcd for C_9_H_7_NOS: C, 61.00; H, 3.98; N, 7.90. Found: C, 60.95; H, 3.97; N, 7.79.

*1-Hydroxyquinoline-2-(1H)-thione* (**g**). Yield: 89%. ^1^H NMR (500 MHz, DMSO-d_6_): *δ =* 12.14 (s, 1H, N–O*H*), 7.94–7.90 (m, 2H, Ar–*H*), 7.85 (d, 1H, *J* = 9.1 Hz, Ar–*H*), 7.82–7.77 (m, 1H, Ar–*H*), 7.52 (d, 1H, *J* = 9.1 Hz, Ar–*H*), 7.50–7.46 (m, 1H, Ar–*H*). ESI-HRMS (CH_3_CN) *m*/*z* for [M + H]^+^ (found (calcd)): 178.0324 (178.0321). Anal. Calcd for C_9_H_7_NOS: C, 61.00; H, 3.98; N, 7.90. Found: C, 61.23; H, 3.81; N, 7.82.

*2-Hydroxyisoquinoline-3-(2H)-thione* (**h**). Yield: 88%. ^1^H NMR (500 MHz, DMSO-d_6_): *δ =* 12.97 (bs, 1H, N–O*H*), 9.73 (s, 1H, Ar–*H*), 8.09 (s, 1H, Ar–*H*), 7.95 (d, 1H, *J* = 8.0 Hz, Ar–*H*), 7.75–7.66 (m, 2H, Ar–*H*), 7.50–7.45 (m, 1H, Ar–*H*). ESI-HRMS (CH_3_CN) *m/z* for [M + H]^+^ (found (calcd)): 178.0306 (178.0321).

General procedure for **1f**–**h**. A mixture of RuCym precursor (1 mol. equiv.), ligand **f**–**h** (2.2 mol. equiv.), and NaOMe (1.9 mol, equiv.) was stirred at room temperature in DCM overnight. Next day the solvent was evaporated under the reduced pressure on a rotary evaporator followed by the purification of the crude product by column chromatography on silica gel, eluting with 2% MeOH/DCM. After evaporating the mobile phase under reduced pressure, an oily residue was obtained. The residue was redissolved in DCM (approximately 10 mL) and the solvent was again evaporated to ensure total removal of MeOH. The oily product was again dissolved in 1–2 mL of DCM and the addition of the cold *n*-heptane (10–15 mL) resulted in the complex precipitation. If the complexes did not precipitate, the solvents were partly evaporated on the rotary evaporator to remove exceeded DCM and/or ultrasonic bath was used to ease the precipitation of the complexes. The suspension was left to stand for 30 min, after which the product was filtered under reduced pressure and washed with cold *n*-heptane. Obtained red solids were dried at 45 °C overnight.

*[(η^6^-p-Cymene)Ru(2-hydroxyisoquinoline-1-(2H)-thionato)Cl]* (**1f**). Yield: 71%. ^1^H NMR (500 MHz, CDCl_3_): *δ =* 8.63–8.59 (m, 1H, Ar–*H* f), 7.92 (d, 1H, *J* = 7.3 Hz, Ar–*H* f), 7.65–7.55 (m, 3H, Ar–*H* f), 7.11 (d, 1H, *J* = 7.3 Hz, Ar–*H* f), 5.53 (d, 2H, *J* = 6.1 Hz, Ar–*H* cym), 5.33 (d, 2H, *J* = 4.5 Hz, Ar–*H* cym), 2.85 (hept, 1H, *J* = 6.9 Hz, Ar–C*H*(CH_3_)_2_ cym), 2.27 (s, 3H, Ar–C*H*_3_ cym), 1.27 (d, 6H, *J* = 6.9 Hz, Ar–CH(C*H*_3_)_2_ cym). IR selected bands (cm^−1^, ATR): 3047, 2959, 1550, 1343, 1175, 939, 818, 772, 752, 667. UV-Vis (*λ* (nm) (*ε* (L mol^−1^ cm^−1^)) *c* = 5 × 10^−5^ M, MeOH): 304 (12030), 346 (sh) (5302), 392 (sh) (2486), 487 (600). ESI-HRMS (CH_3_CN) *m/z* for [M − Cl]^+^ (found (calcd)): 412.0319 (412.0309). Anal. Calcd for C_19_H_20_ClNORuS: C, 51.06; H, 4.51; N, 3.13. Found: C, 50.92; H, 4.47; N, 3.08.

*[(η^6^-p-Cymene)Ru(1-hydroxyquinoline-2-(1H)-thionato)Cl]* (**1g**). Yield: 64%. ^1^H NMR (500 MHz, CDCl_3_): *δ =* 8.54 (d, 1H, *J* = 8.5 Hz, Ar–*H* g), 7.69–7.63 (m, 2H, Ar–*H* g), 7.46–7.39 (m, 3H, Ar–*H* g), 5.57 (s, 2H, Ar–*H* cym), 5.32 (s, 2H, Ar–*H* cym), 2.86 (hept, 1H, *J* = 6.9 Hz, Ar–C*H*(CH_3_)_2_ cym), 2.29 (s, 3H, Ar–C*H*_3_ cym), 1.30 (d, 6H, *J* = 6.9 Hz, Ar–CH(C*H*_3_)_2_ cym). IR selected bands (cm^−1^, ATR): 3048, 2957, 1612, 1311, 1140, 905, 827, 764, 659, 528. UV-Vis (*λ* (nm) (*ε* (L mol^−1^ cm^−1^)) *c* = 5 × 10^−5^ M, MeOH): 280 (19434), 343 (6148), 417 (sh) (2404), 510 (sh) (568). ESI-HRMS (CH_3_CN) *m/z* for [M − Cl]^+^ (found (calcd)): 412.0314 (412.0309). Anal. Calcd for C_19_H_20_ClNORuS: C, 51.06; H, 4.51; N, 3.13. Found: C, 50.87; H, 4.21; N, 3.11.

*[(η^6^-p-Cymene)Ru(2-hydroxyisoquinoline-3-(2H)-thionato)Cl]* (**1h**). Yield: 49%. ^1^H NMR (500 MHz, CDCl_3_): *δ =* 8.75 (s, 1H, Ar–*H* h), 7.77 (s, 1H, Ar–*H* h), 7.55 (d, 1H, *J* = 8.4 Hz, Ar–*H* h), 7.47 (d, 2H, *J* = 3.7 Hz, Ar–*H* h), 7.33–7.28 (m, 1H, Ar–*H* h), 5.50 (d, 2H, *J* = 5.5 Hz, Ar–*H* cym), 5.29 (s, 2H, Ar–*H* cym), 2.88 (hept, 1H, *J* = 7.0 Hz, Ar–C*H*(CH_3_)_2_ cym), 2.28 (s, 3H, Ar–C*H*_3_ cym), 1.31 (d, 6H, *J* = 7.0 Hz, Ar–CH(C*H*_3_)_2_ cym). IR selected bands (cm^−1^, ATR): 3052, 2956, 1445, 1310, 1104, 879, 815, 742, 630, 464. UV-Vis (*λ* (nm) (*ε* (L mol^−1^ cm^−1^)) *c* = 5 × 10^−5^ M, MeOH): 313 (18644), 369 (sh) (4776), 484 (sh) (808). ESI-HRMS (CH_3_CN) *m/z* for [M − Cl]^+^ (found (calcd)): 412.0312 (412.0309). Anal. Calcd for C_19_H_20_ClNORuS: C, 51.06; H, 4.51; N, 3.13. Found: C, 51.00; H, 4.62; N, 3.12.

### 3.4. Cholinesterase Inhibition Assay

Cholinesterase activities were measured by the Ellman method [49] adapted for microtiter plates, as described in Ristovski et al. [23]. Stock solutions of the prepared ruthenium compounds (1 mg/mL) or neostigmine bromide (Sigma-Aldrich, St. Louis, MO, USA) as a positive control were prepared in 100% ethanol and progressively diluted in 100 mM potassium phosphate buffer (pH 7.4) to a final volume of 50 µL. Then, 1 mM acetylthiocholine chloride and 0.5 mM 5,5′-dithiobis-2-nitrobenzoic acid were dissolved in the same buffer and added to the wells of the microtiter plates to the final volume of 150 μL. Electric eel acetylcholinesterase (eeAChE) or horse serum BChE (hsBuChE) (both Sigma, St. Louis, MO, USA) were dissolved in the same buffer to 0.0075 U/mL. Fifty μL of each of the cholinesterases was added to start the reactions, which were followed spectrophotometrically at 405 nm and 25 °C for 5 min using a kinetic microplate reader (Dynex Technologies, Chantilly, VA, USA). Blank reactions without the inhibitors were run in the presence of the appropriate dilution of ethanol. Every measurement was repeated at least three times. For determination of the inhibitory constants, the kinetics were monitored using three different final substrate concentrations (0.125, 0.25, 0.5 mM). The inhibitory constants were determined only for the compounds that showed an IC_50_ <100 μM.

### 3.5. Computational Details

Calculations were carried out on a PC running at 3.4 GHz on an Intel i7 2600 quad core processor with 8 GB RAM and 1 TB hard disk, with Windows 7 Home Premium 64-bit SP1 as the operating system. The ligands structure **1a**–**c** and **1e**–**g** derive from X-ray crystallographic data, whereas the structures of compounds **1d** and **1h** were obtained by quantum chemical calculations using the Gaussian 03W revision E.01 package program set [50]. Restricted mode was used and performed in vacuo for geometry optimization. The basis set of choice was SDD [51]. The gradient-corrected DFT with the three-parameter hybrid functional (B3) [52] for the exchange part and the Lee–Yang–Parr correlation function [53] were utilized and the optimized structural parameters were employed in the vibrational energy calculations at the same DFT levels to characterize all stationary points as minima. For each optimized structure, no imaginary wavenumber modes were obtained, proving that a local minimum on the potential energy surface was found. All the structures were saved in pdb extension. The AutoDock Tools (ADT) package version 1.5.6rc3 [54] was used to generate the docking input files and to analyze the docking results, with Autodock 4.2.6 [55] used for the docking calculations. The AChE crystallographic structure was downloaded from the Protein Data Bank (PDB; http://www.pdb.org/). The structure of *Torpedo californica* AChE complexed with 12-amino-3-chloro-6,7,10,11-tetrahydro-5,9-dimethyl-7,11-methanocycloocta[b]quinolin-5-ium (6G1V) was determined by X-ray crystallography, with a resolution of 1.8 Å [56]. The structure was modified as follows: the ligand and all of the crystallization water molecules were removed, with the file saved in pdb extension; all hydrogen atoms were added using AutoDock Tools (ADT), and the Gasteigere Marsili charges were calculated, with the resulting file saved in pdbqt extension; rotatable bonds were defined for each ligand molecule. For the docking calculation, and for 6G1V, a grid box of 40 × 40 × 40 number of points in the x, y, and z directions was created, with spacing of 0.375 Å and centered at x = 3.655, y = −4.537, z = 20.935. The following parameters were used in docking calculation: number of independent Genetic Algorithm (GA) runs = 10; population size = 150; maximum number of evaluation = 2,500,000; maximum number of generations 27,000; maximum number of top individuals that automatically survive = 1; rate of gene mutation = 0.01; rate of crossover = 0.8; genetic algorithm crossover mode = twopt; Mean of Cauchy distribution for gene mutation = 0.0; Variance of Cauchy distribution for gene mutation = 1.0; Number of generations for picking worst individual = 10. To validate the accuracy of the calculation, the original ligand was re-docked; visual inspection of the data showed a very tight overlap. The results are expressed as the energy associated to each ligand-enzyme complex in terms of the Gibbs free energy values, with a standard error in AutoDock score function evaluated as 2.5 Kcal/mol. The visual ligand–enzyme interactions were displayed using the server Protein-Ligand Interaction Profiler (PLIP) [57]. ADME predictions were performed using the online server Swiss-ADME [58].

## 4. Conclusions

In this study we have prepared eight organoruthenium(II) chlorido complexes **1a**–**h** with pyrithione **a**, its methyl-substituted ligands **b**–**e**, and bicyclic aromatic analogues **f**–**h**. New compounds **1f**–**h** have been physicochemically characterized by ^1^H NMR, IR, UV-Vis spectroscopy, HRMS, and CHN elemental analysis. Additionally, crystal structures of the complexes **1f** and **1g** have been obtained, which have revealed pseudo-octahedral geometry with bidentately bound *O,S*-ligands to ruthenium(II) core and monodentate chlorido ligand completing the coordination sphere. For the best two performing complexes, **1e** and **1g** against eeAChE and hsBuChE, NMR stability tests in different D_2_O solutions were performed and showed that complexes remained stable. Furthermore, an ESI-MS study has confirmed that chlorido complexes stay stable in aqueous solutions with the same [M–Cl]^+^ peak observed after immediate dissolution of the complexes in water and after three days for all tested compounds. After confirming the stability, which is a prerequisite for further biological assays, ligands **a**–**h** and their organoruthenium(II) complexes **1a**–**h** were evaluated as potential inhibitors against eeAChE and hsBuChE. Ligands showed no activity towards these enzymes, whereas notable differences in the activity have been achieved by various methyl-substituted and bicyclic aromatic pyrithione complexes in comparison to the referenced pyrithione complex **1a**. Taking into consideration compounds **1b**–**e**, complexes **1d** and **1e** with methyl substituents at the position 5 and 6 expressed improved inhibition against eeAChE and hsBuChE in comparison to **1a**. Furthermore, after the aromatic ring extension at these two positions forming 1-hydroxyquinoline-2-(1*H*)-thione ligand **g**, the activity of the complex **1g** towards both cholinesterases was further improved. Importantly, complex **1g** expresses the best inhibition on eeAChE as well as hsBuChE, which is an advantage, as AChE plays important role at the early stages of the development of AD, continued by the increased activity of BuChE. Moreover, docking calculations confirmed complex **1g** to possess the lowest calculated Gibbs free energy value and suggested H-bonding, π-stackings, and many other hydrophobic interactions with TcAChE. To conclude, binding of pyrithione-type ligands to ruthenium(II) resulted in potent metal inhibitors against tested cholinesterases, where tuning of the activity can be achieved by enhanced lipophilicity and certain positional changes on pyrithione scaffold.

## Figures and Tables

**Table 1:** Inhibition of electric eel acetylcholinesterase (eeAChE) and horse serum butyrylcholinesterase (hsBuChE) by non-complexed ligands and their chlorido complexes tested.

Compound	eeAChE		hsBuChE	
	IC_50_ (µM) ^1^	*Ki* (µM) ^2^	IC_50_ (µM) ^1^	*Ki* (µM) ^2^
**a**	/	/	/	/
**b**	/	/	/	/
**c**	/	/	/	/
**d**	/	/	/	/
**e**	/	/	>100	/
**f**	/	/	/	/
**g**	/	/	/	/
**h**	>100	/	53	n.d.
**1a**	7.8 ± 0.8	15.1	2.3 ± 0.1	0.5
**1b**	10.5 ± 0.7	9.7	1.2 ± 0.02	0.4
**1c**	9.5 ± 1.1	8.5	2.7 ± 0.8	0.9
**1d**	6.6 ± 0.5	9.7	1.9 ± 0.4	0.4
**1e**	5.1 ± 0.4	4.9	0.7 ± 0.1	3.4
**1f**	8.1 ± 0.5	12.3	1.3 ± 0.1	0.4
**1g**	**4.9 ± 0.1**	5.6	**0.2 ± 0.05**	0.2
**1h**	14.3 ± 2.0	20.1	0.7 ± 0.1	0.2
Neostigmine bromide	4.3 ± 0.8	/	37.3 ± 4.2	/

^1^ IC_50_ values determined as concentration inducing 50% inhibition of enzyme activity; data are mean ± SEM of three independent measurements. Underlined are IC_50_ values of the complexes that are lower than that of the referenced **1a**. The results of the most active compound **1g** are in bold. ^2^ *K_i_* not determined for the compounds with IC_50_ value >100 μM, or with no inhibitory activity; / = no activity. n.d. = not determined.

**Table 2:** Data from docking calculation of the compounds **1a**–**h** with TcAChE.

Compound ^1^	Interactions				
	ΔE (Kcal/mol)	H-Bond ^2^	Hydrophobic ^2^	π-Stacking ^2^	Salt Bridge ^2^
**1a**	−7.55	-	Y70 (3.43); Y121(3.47) F330(3.57; 3.14)	W84 (4.38; 3.73)	-
**1b**	−7.16	S122 (3.09)	D72 (3.82); F330 (3.34)	W84 (4.04)	-
**1c**	−7.02	-	Y70 (3.60); D72 (3.62) N85 (3.96); F330 (3.92; 3.35); Y334 (3.38)	W84 (4.62) F330 (5.09)	-
**1d**	−8.60	-	D72 (3.62); W84 (3.39; 3.78) F330 (3.66); Y334 (3.39); I444 (3.96) W432 (3.77); Y442 (3.67) I444 (372)	-	-
**1e**	−6.73	-	D72 (3.99); W 84 (3.37; 3.41) F290 (3.73); F330 (3.13) F331 (3.58)	-	-
**1f**	−8.31	-	D72 (3.66); W84 (3.55; 3.87); F330 (3.23; 3.16); L333 (3.94) Y334 (2.95; 3.27); W432 (3.08) I439 (3.52)	W84 (3.81) F330 (3.63)	-
**1g**	−9.86	Y121 (2.47)	Y70 (3.02); D72 (3.40) Y121 (3.46); F290 (3.51)	F330 (4.35) F331 (5.34)	-
**1h**	−8.28	-	W84 (3.57; 3.94; 3.97) F330 (3.63); W432 (3.80) Y442 (3.51)	W84 (3.61) F330 (3.84)	-
**neostigmine bromide**	−7.03	Y121 (2.93)	Y70 (3.72); W279 (3.36) F330 (3.57)	-	D72 (3.95)

^1^ Structures **1d** and **1h** minimized by using Gaussian 09 before docking calculation. ^2^ Distance (in Å) reported in brackets; - = not present.

## Supplementary Materials

Supplementary materials can be found at https://www.mdpi.com/1422-0067/21/16/5628/s1.

## Abbreviations

ACh	acetylcholine
AChE	acetylcholinesterase
AD	Alzheimer’s disease
Aβ	amyloid-β
BuChE	butyrylcholinesterase
ChE	cholinesterase
DCM	dichloromethane
DMSO	dimethyl sulfoxide
ee	electric eel
ESI-MS	electrospray ionization mass spectrometry
ESI-HRMS	high-resolution electrospray ionization mass spectrometry
hs	horse serum
MeOH	methanol
NMR	nuclear magnetic resonance
RuCym	ruthenium precursor [Ru(*p*-cymene)Cl_2_]_2_
Tc	*Torpedo californica*
TLC	thin layer chromatography
