# Supplementary material for: Structural Isomerism and Enhanced Lipophilicity of Pyrithione Ligands of Organoruthenium(II) Complexes Increase Inhibition on AChE and BuChE
Source: Int J Mol Sci. 2020 Aug 6;21(16):5628. doi: 10.3390/ijms21165628 (PMC7460603; doi:10.3390/ijms21165628)
Supplement: Supplementary file 1 [file ijms-21-05628-s001.pdf]

Structural Isomerism and Enhanced Lipophilicity of Pyrithione Ligands of Organoruthenium(II) Complexes Increase Inhibition on AChE and BuChE

Jerneja Kladnik [1], Samuel Ristovski [2], Jakob Kljun [1], Andrea Defant [3], Ines Mancini [3], Kristina Sepčič [2,*] and Iztok Turel [1,*]

[1] Faculty of Chemistry and Chemical Technology, University of Ljubljana, Večna pot 113, SI-1000 Ljubljana, Slovenia; jerneja.kladnik@fkkt.uni-lj.si (J.Kla.), jakob.kljun@fkkt.uni-lj.si (J.Klj.), iztok.turel@fkkt.uni-lj.si (I. T.)

[2] Department of Biology, Biotechnical Faculty, University of Ljubljana, Jamnikarjeva 101, 1000 Ljubljana, Slovenia; samuel.ritovski@gmail.com (S.R.), kristina.sepcic@bf.uni-lj.si (K.S.)

[3] Department of Physics, Bioorganic Chemistry Laboratory, University of Trento, via Sommarive, 14 I-38123 Povo-Trento, Italy; andrea.defant@unitn.it (A.D.), ines.mancini@unitn.it (I.M.)

* Correspondence: kristina.sepcic@bf.uni-lj.si (K.S.), iztok.turel@fkkt.uni-lj.si (I.T.)

Table of Contents

1. Synthesis of the ligands **f-h** and their complexes **1f-h**........2
2. Single crystal X-ray diffraction........3
3. NMR stability........5
4. NMR spectra........8
5. IR spectra........17
6. Additional computational data........20
7. ADME prediction data........21

1. **Synthesis of the ligands f-h and their complexes 1f-h.**

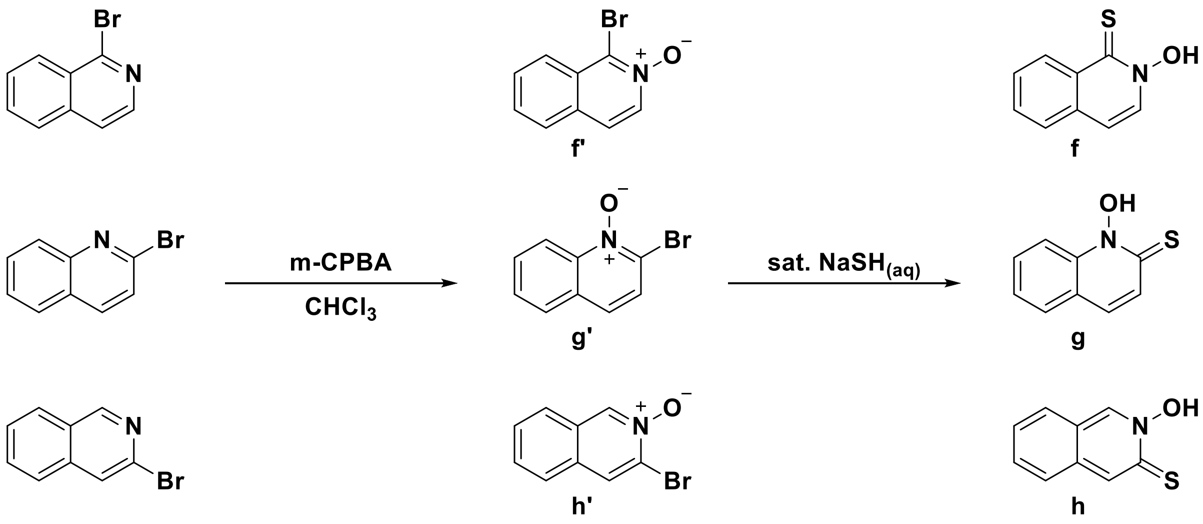

Scheme S1. General scheme of *N*-oxidation and thiolation for the ligands **f-h**.

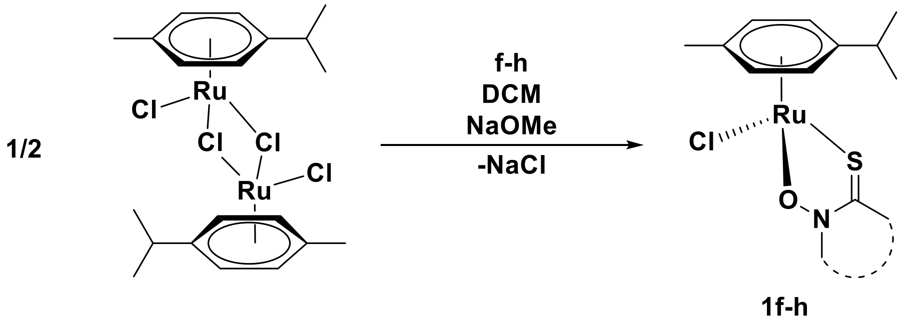

Scheme S2. General scheme of the synthesis for the organoruthenium(II) complexes **1f-h**.

2. Single crystal X-ray diffraction.

Table S1. Crystallographic data for the compounds **1f**, and **1g**.

Compound	**1f**	**1g**
Empirical formula	$C_{19}H_{20}ClNORuS$	$C_{19}H_{20}ClNORuS$
Formula weight	446.94	446.94
Temperature/K	150.00(10)	150.00(10)
Crystal system	triclinic	monoclinic
Space group	P-1	$P2_1/n$
a/Å	9.3454(4)	12.1593(6)
b/Å	9.7451(5)	10.8164(6)
c/Å	10.3401(5)	14.5652(7)
α/°	91.210(4)	90
β/°	102.660(4)	97.892(5)
γ/°	101.940(4)	90
Volume/Å^3	896.74(8)	1897.47(17)
Z	2	4
ρ_{calc}g/cm^3	1.655	1.565
μ/mm^{-1}	1.145	1.082
F(000)	452.0	904.0
Crystal size/mm^3	0.15 × 0.1 × 0.1	0.10 × 0.05 × 0.05
Radiation	MoKα (λ = 0.71073)	MoKα (λ = 0.71073)
2Θ range for data coll./°	5.548 to 54.968	5.566 to 54.96
Index ranges	$-12 \le h \le 12$, $-12 \le k \le 12$, $-11 \le l \le 13$	$-15 \le h \le 15$, $-13 \le k \le 14$, $-18 \le l \le 18$
Reflections collected	7902	11118
Independent reflections	4112 [R_{int} = 0.0310, R_{sigma} = 0.0484]	4336 [R_{int} = 0.0825, R_{sigma} = 0.0788]
Data/restraints/parameters	4112/0/220	4336/0/220
Goodness-of-fit on F^2	1.047	1.060
Final R indexes [I>=2σ (I)]	R_1 = 0.0321, wR_2 = 0.0661	R_1 = 0.0646, wR_2 = 0.1604
Final R indexes [all data]	R_1 = 0.0414, wR_2 = 0.0710	R_1 = 0.0855, wR_2 = 0.1881
Largest diff. peak/hole / eÅ^{-3}	0.66/-0.59	1.92/-1.65

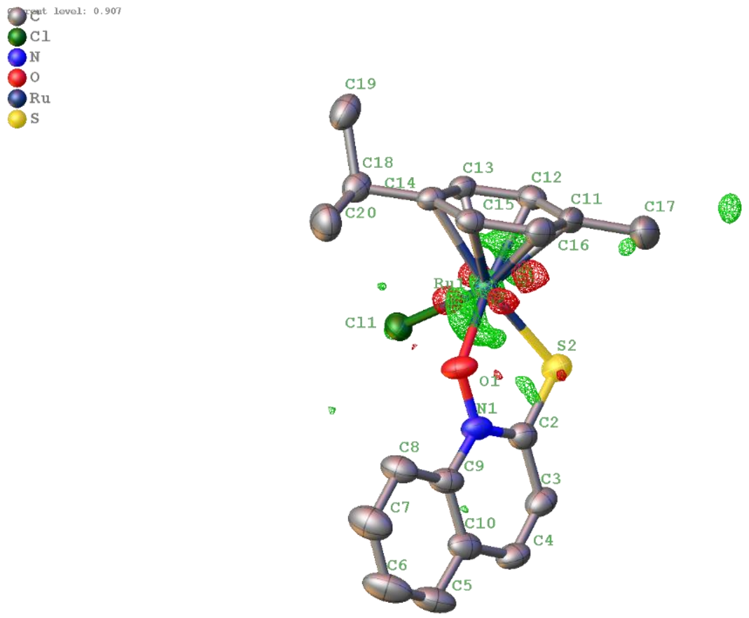

Figure S1. Residual electron density around the ruthenium atom. Red colour denotes peaks, green colour denotes holes.

<u>Comment to the checkcif report.</u> The checkcif report to the crystal structure of compound **1g** presents several alerts arising from issues of X-ray absorption and not ideal crystal quality. All bond lengths and angles as well as thermal ellipsoid parameters are within normal range for organoruthenium complexes. At present this structure is the best we were able to obtain.

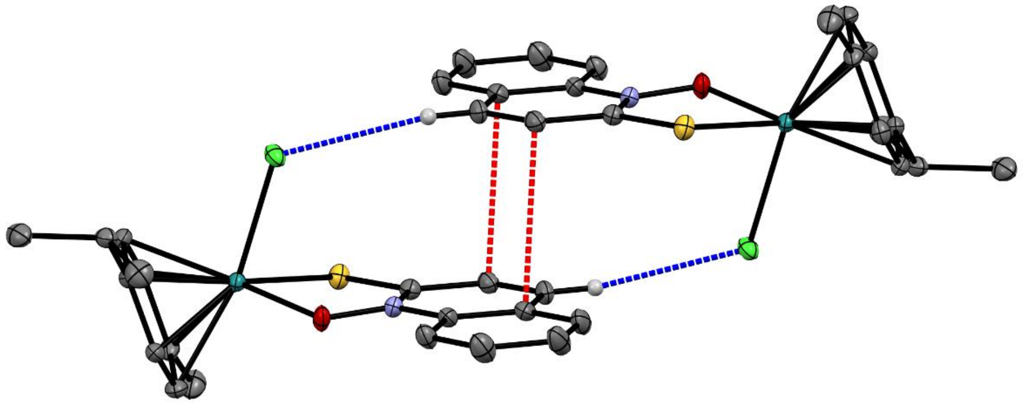

Figure S2: Crystal packing in the compound **1g**. The molecules of the compound **1g** form dimers in solid state. The blue dashed line represents a weak C(Ar)-H···Cl hydrogen bond, the red dashed line shows π-stacking interactions. Thermal ellipsoids are drawn at 20% probability level, H atoms not involved in intramolecular interactions are omitted for better clarity of presentation.

3. NMR stability

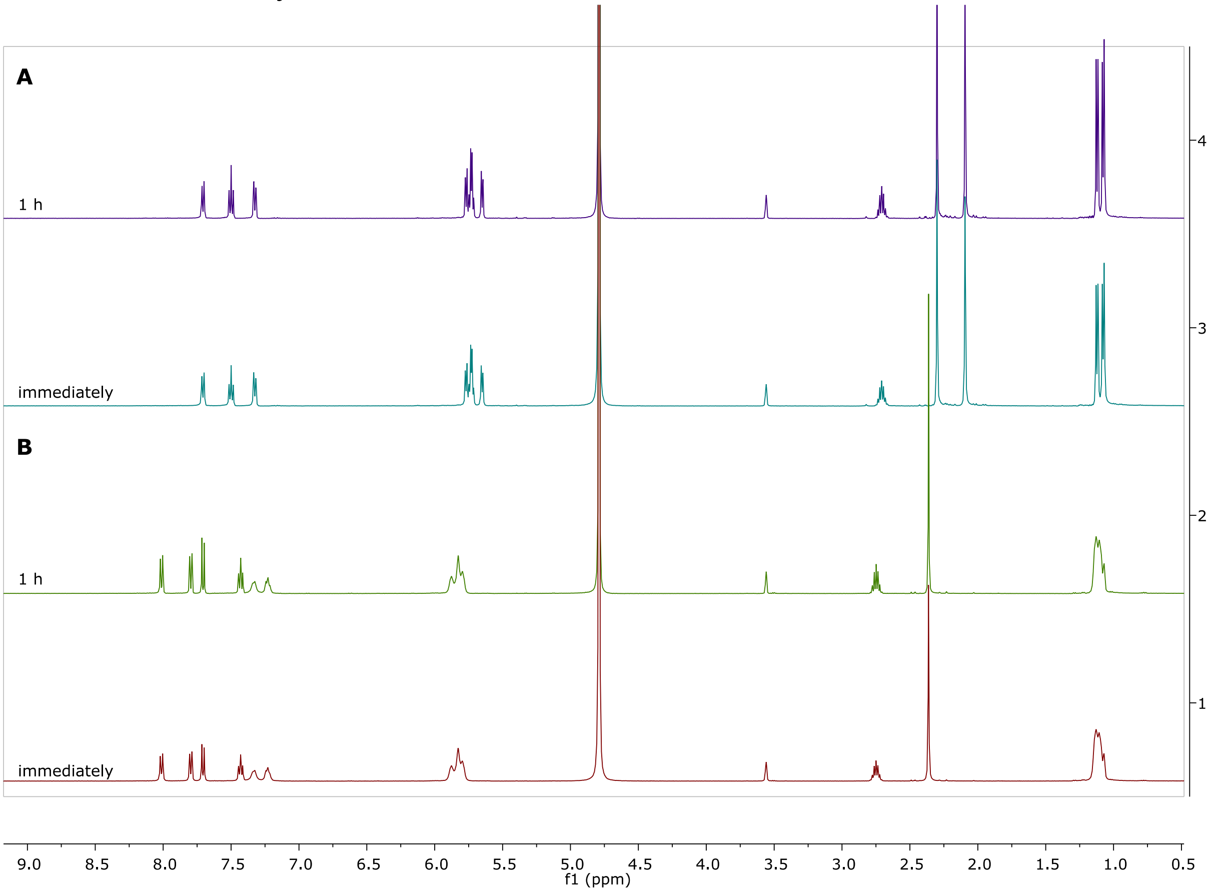

Figure S3: Selected spectra of the chlorido complex **1e** (**A**) and **1g** (**B**) in 6.25% EtOH-d_6/D_2O containing 80.2 mM K_2HPO_4 and 19.8 mM KH_2PO_4, followed by ^{1}H NMR spectroscopy immediately after the dilution of the samples and after 1h.

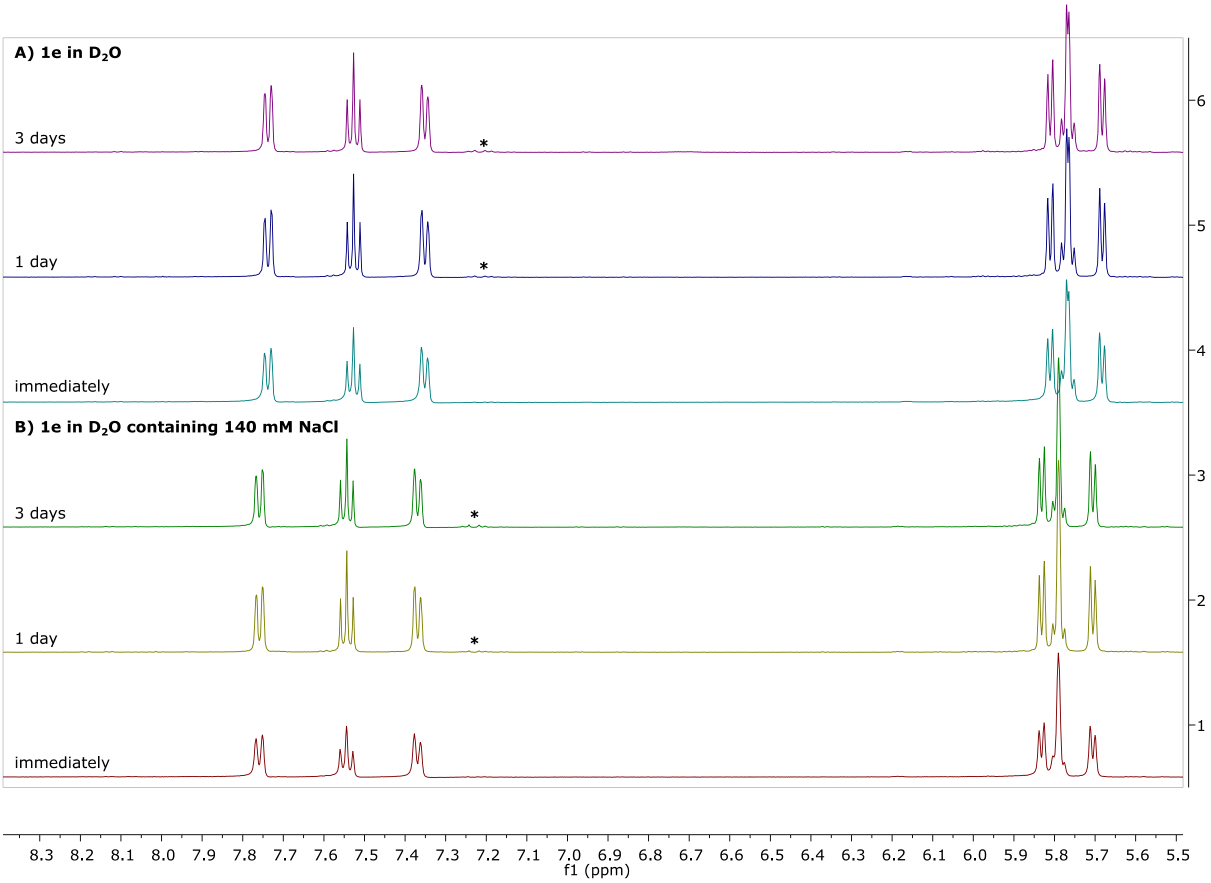

Figure S4: Selected spectra of the chlorido complex **1e** in D_2O (**A**) or in D_2O containing 140 mM NaCl (**B**), followed by ^1H NMR spectroscopy immediately after the dilution, after one and three days. The release of *p*-cymene is labelled with an asterisk (*).

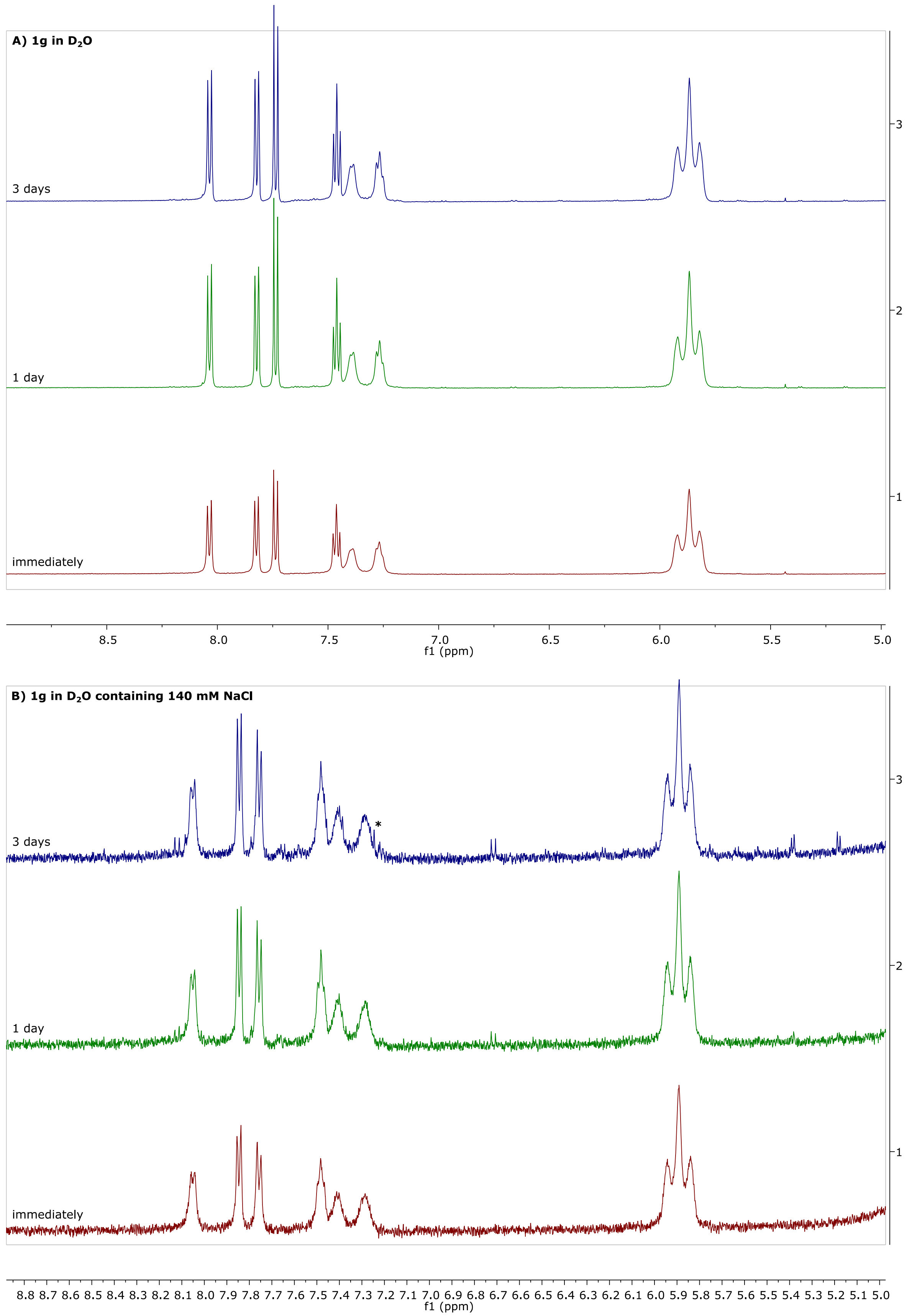

Figure S5: Selected spectra of the chlorido complex **1g** in D_2O (**A**) or in D_2O containing 140 mM NaCl (**B**), followed by ^1H NMR spectroscopy immediately after the dilution, after one and three days. The release of *p*-cymene is labelled with an asterisk (*).

4. NMR spectra

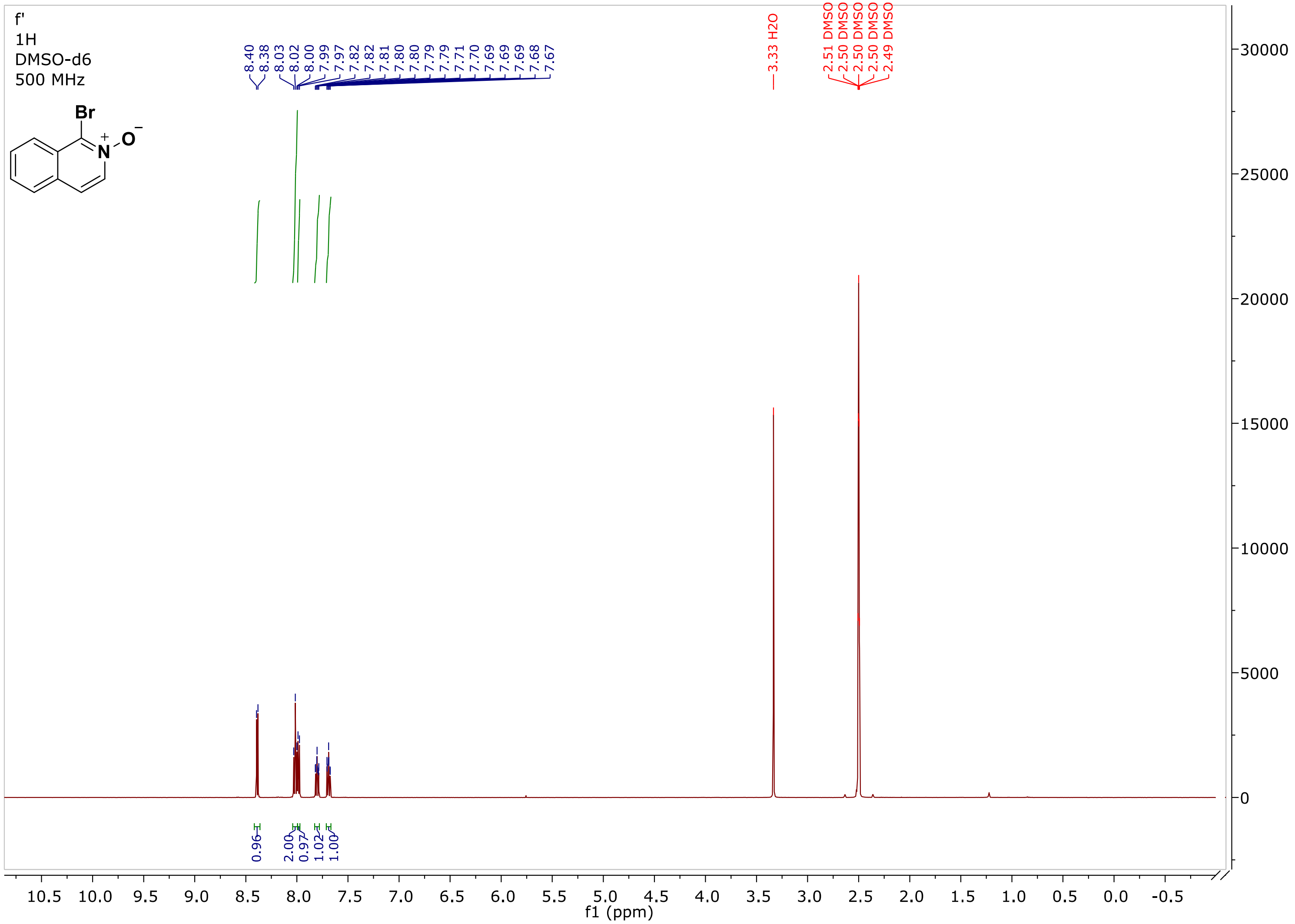

Figure S6: ^{1}H NMR spectrum of **f′**.

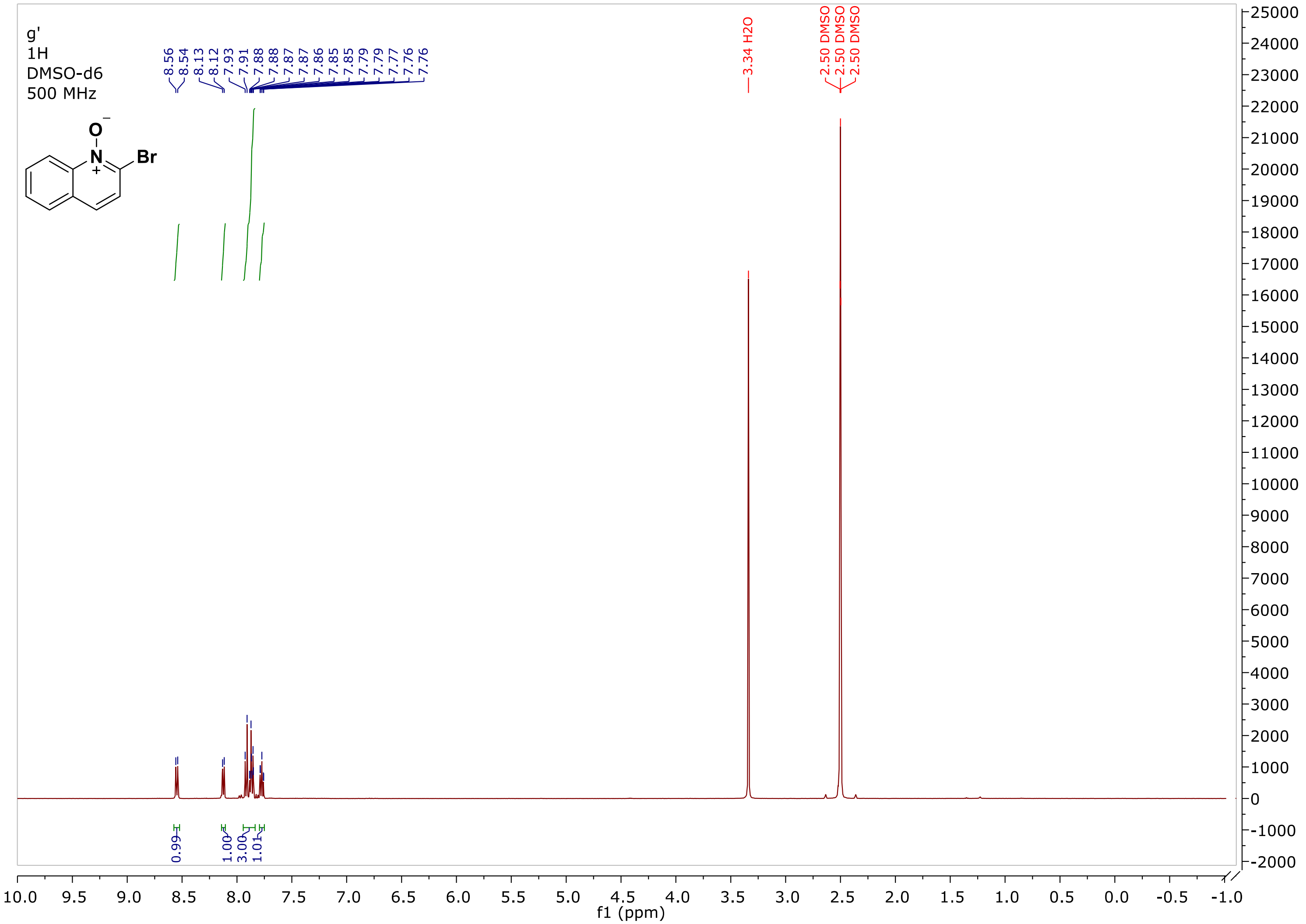

Figure S7: ^{1}H NMR spectrum of **g′**.

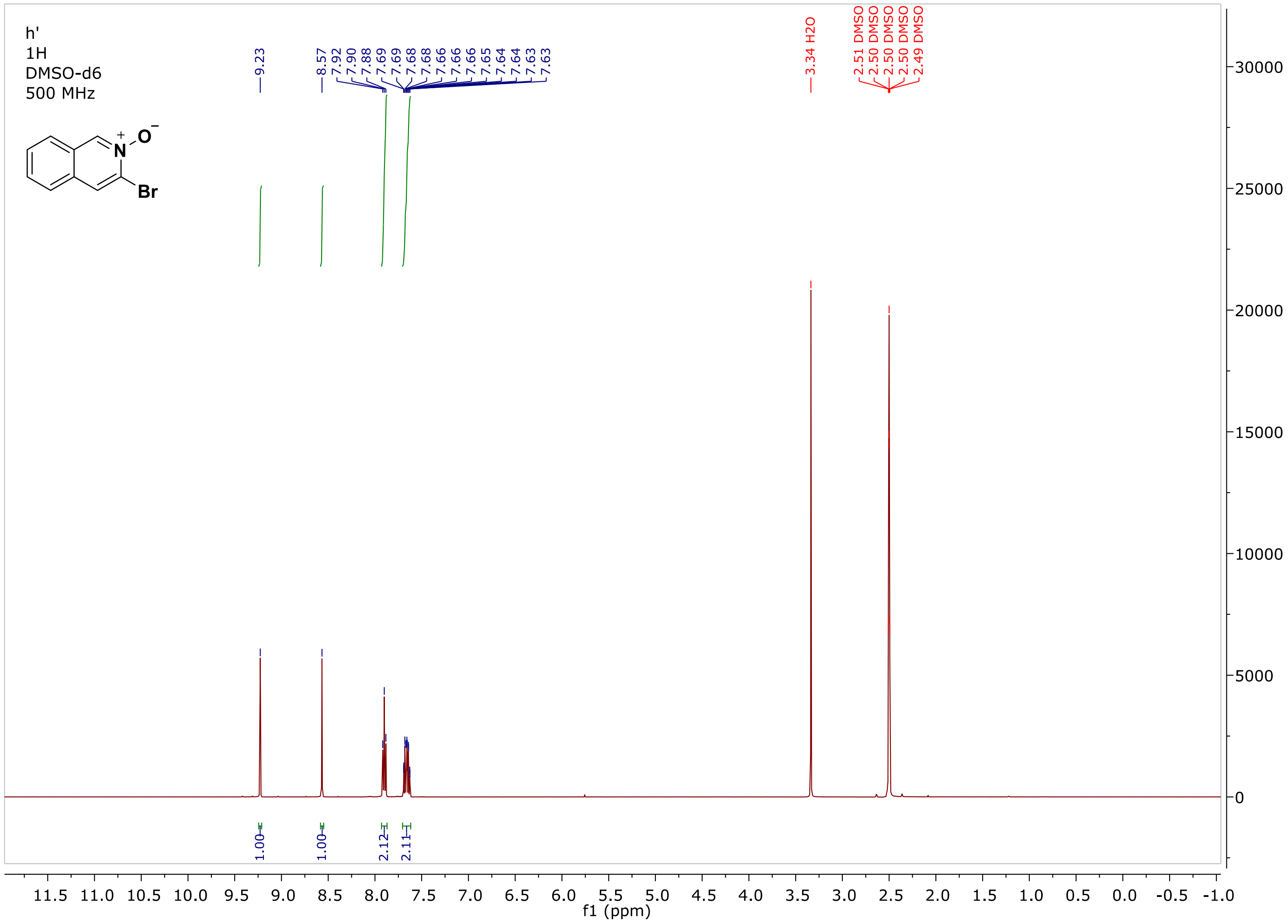

Figure S8: ^{1}H NMR spectrum of **h′**.

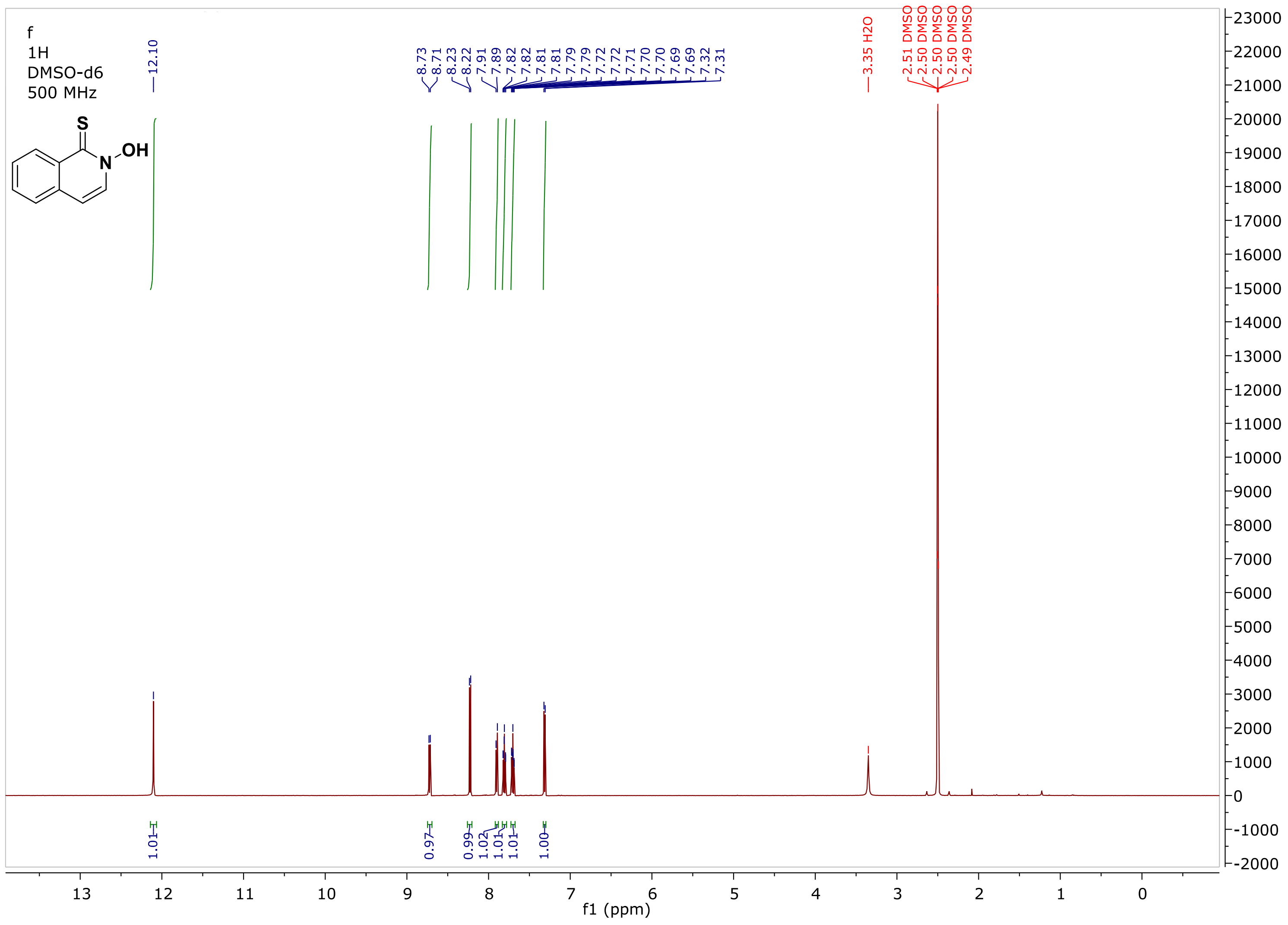

Figure S9: ^{1}H NMR spectrum of **f**.

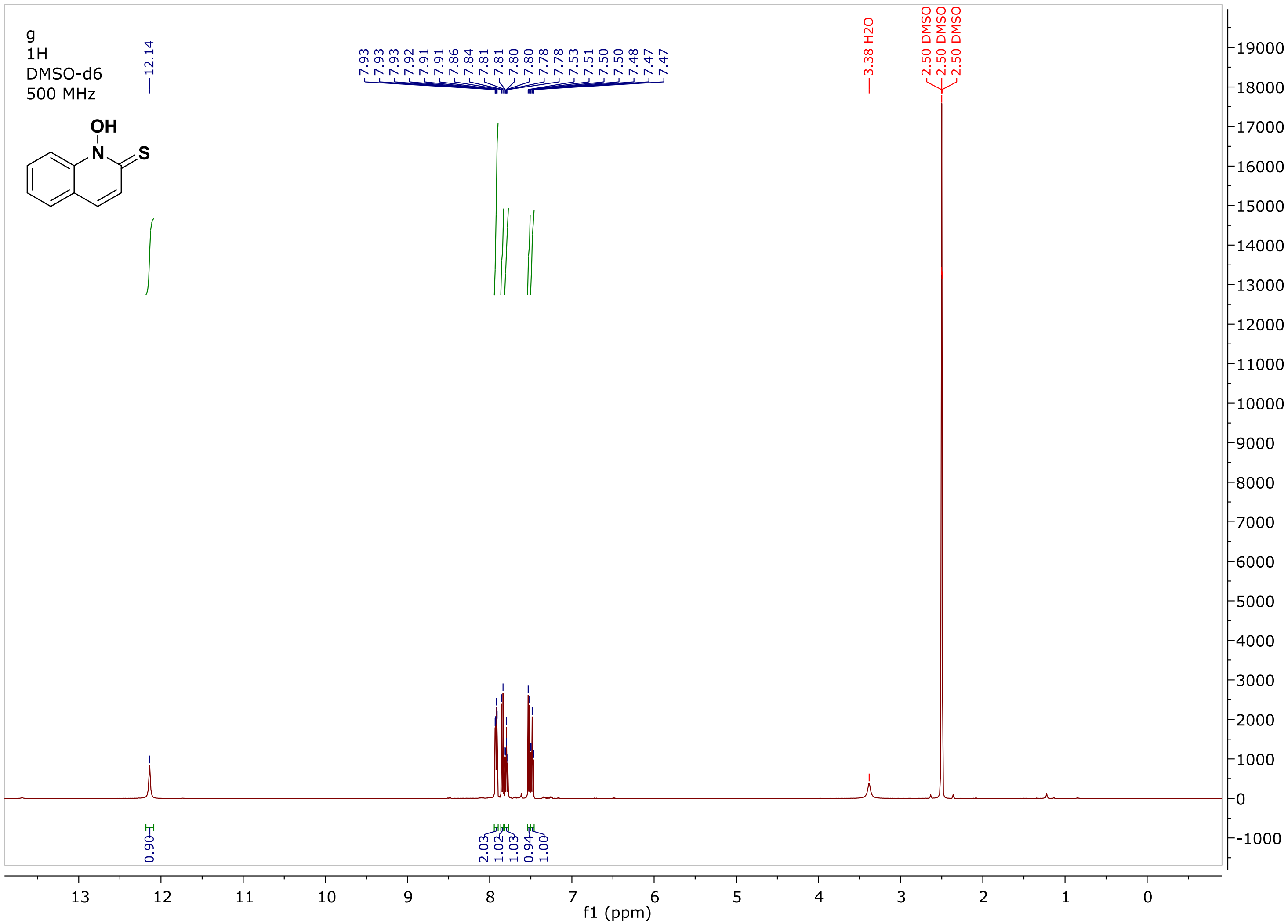

Figure S10: ^{1}H NMR spectrum of **g**.

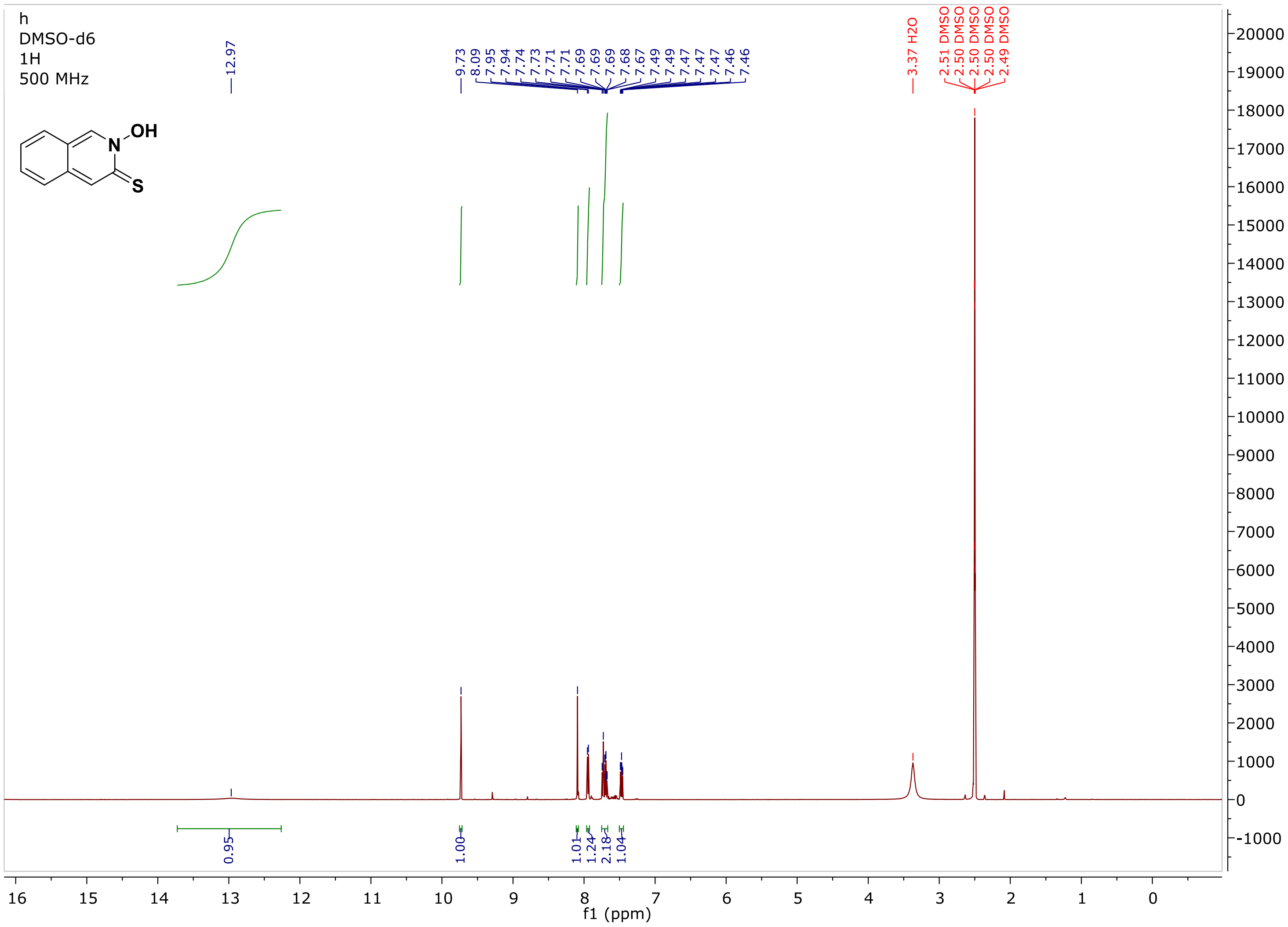

Figure S11: ^{1}H NMR spectrum of **h**.

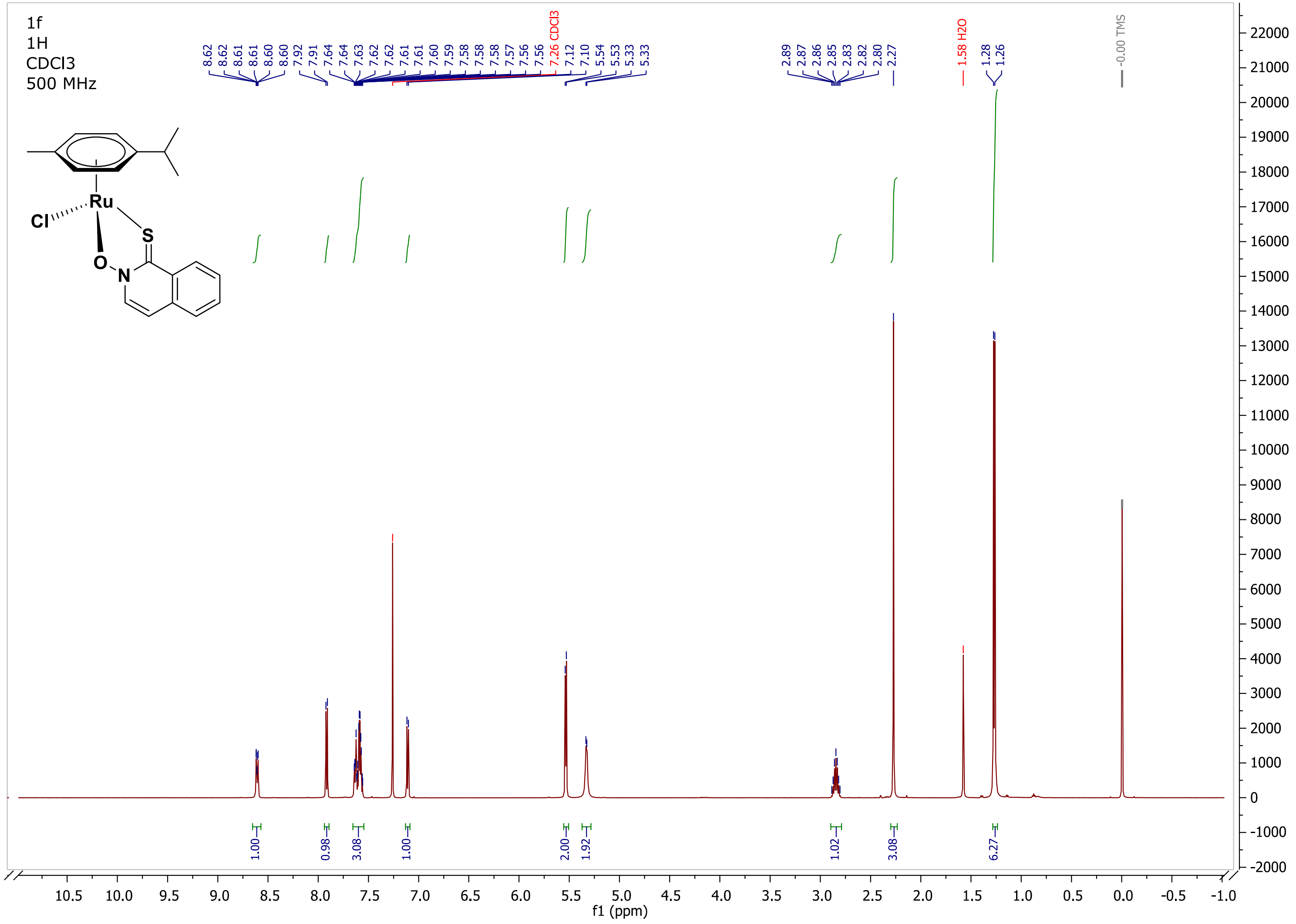

Figure S12: ^{1}H NMR spectrum of **1f**.

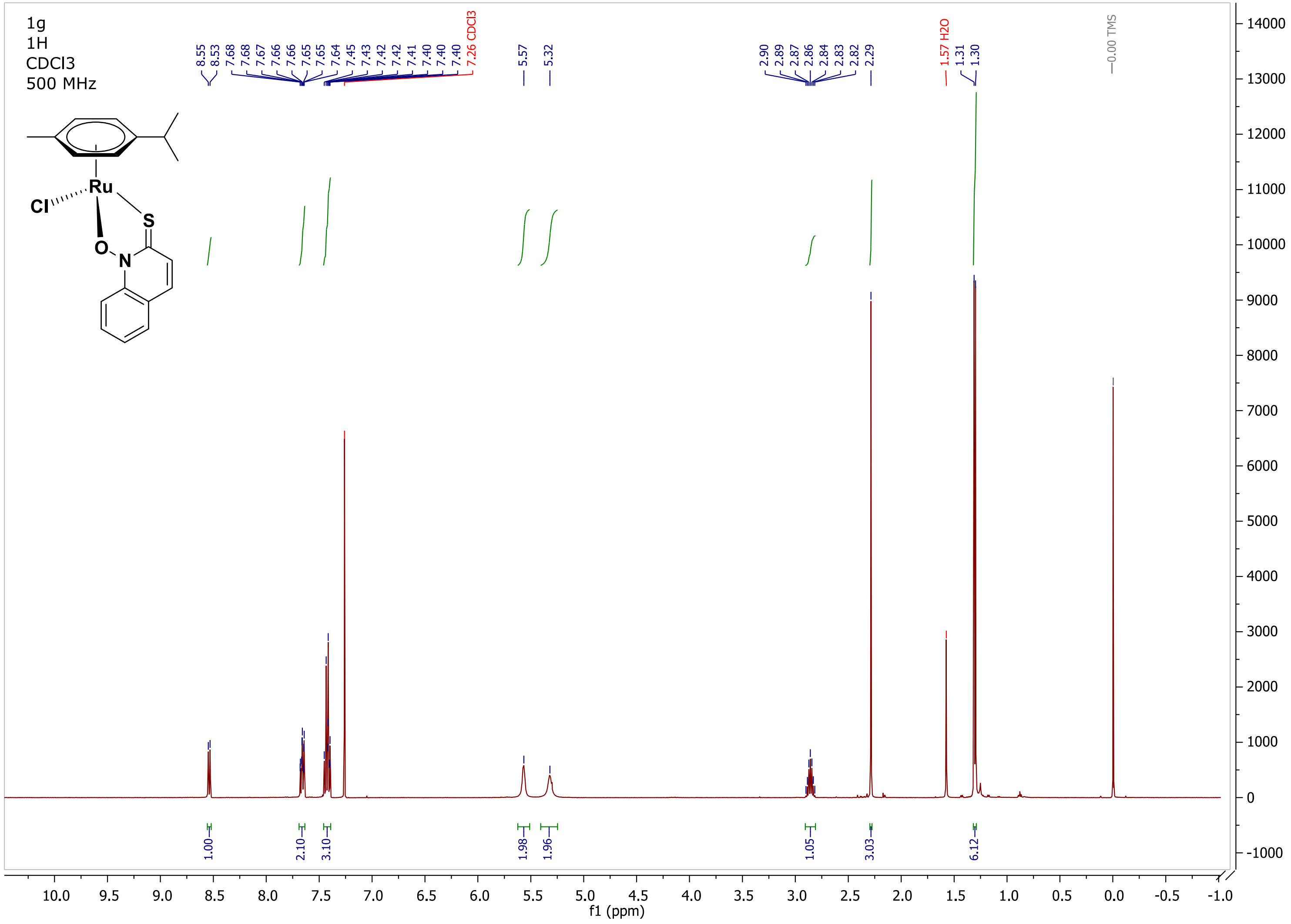

Figure S13: ^{1}H NMR spectrum of **1g**.

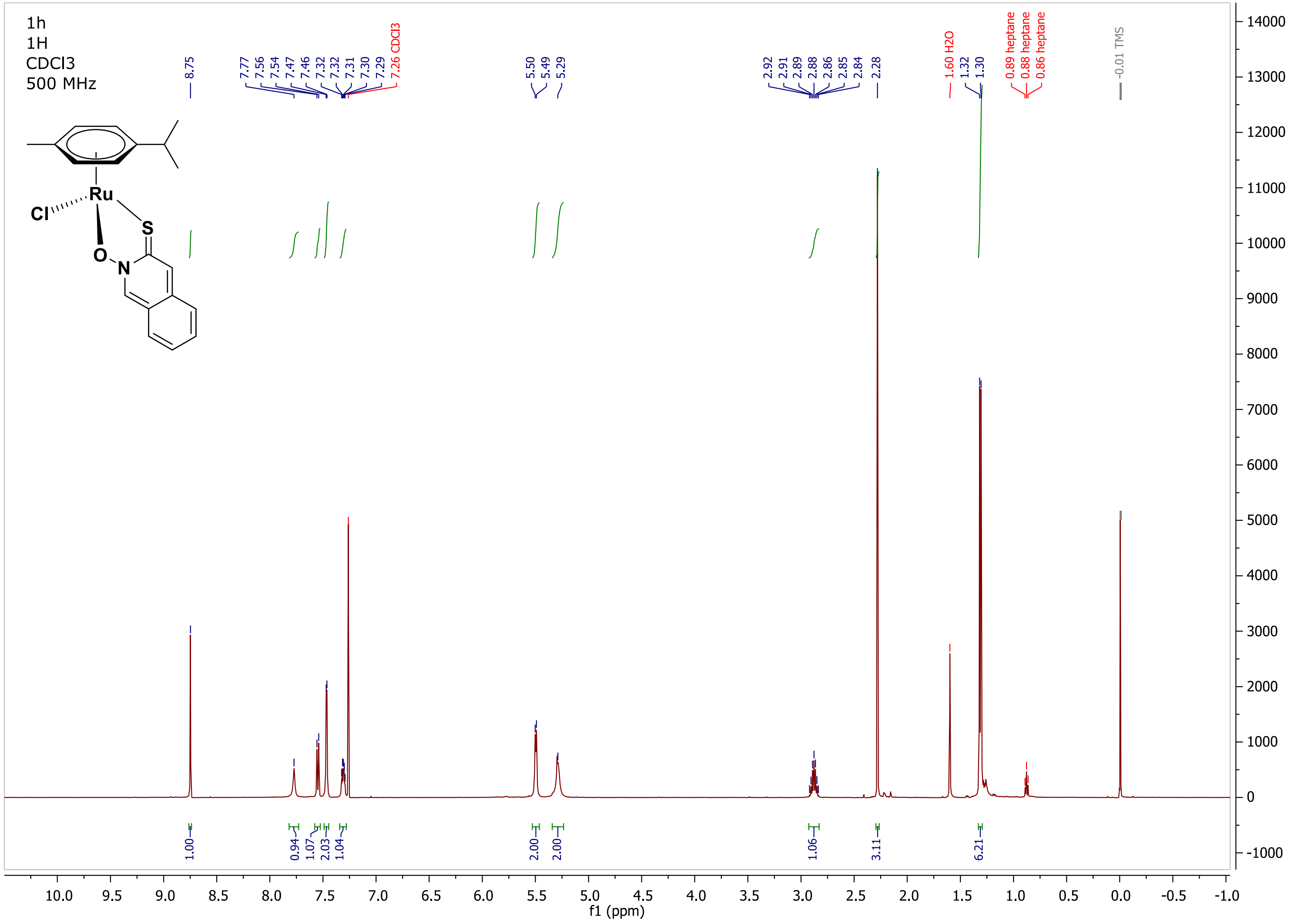

Figure S14: ^{1}H NMR spectrum of **1h**.

5. IR spectra

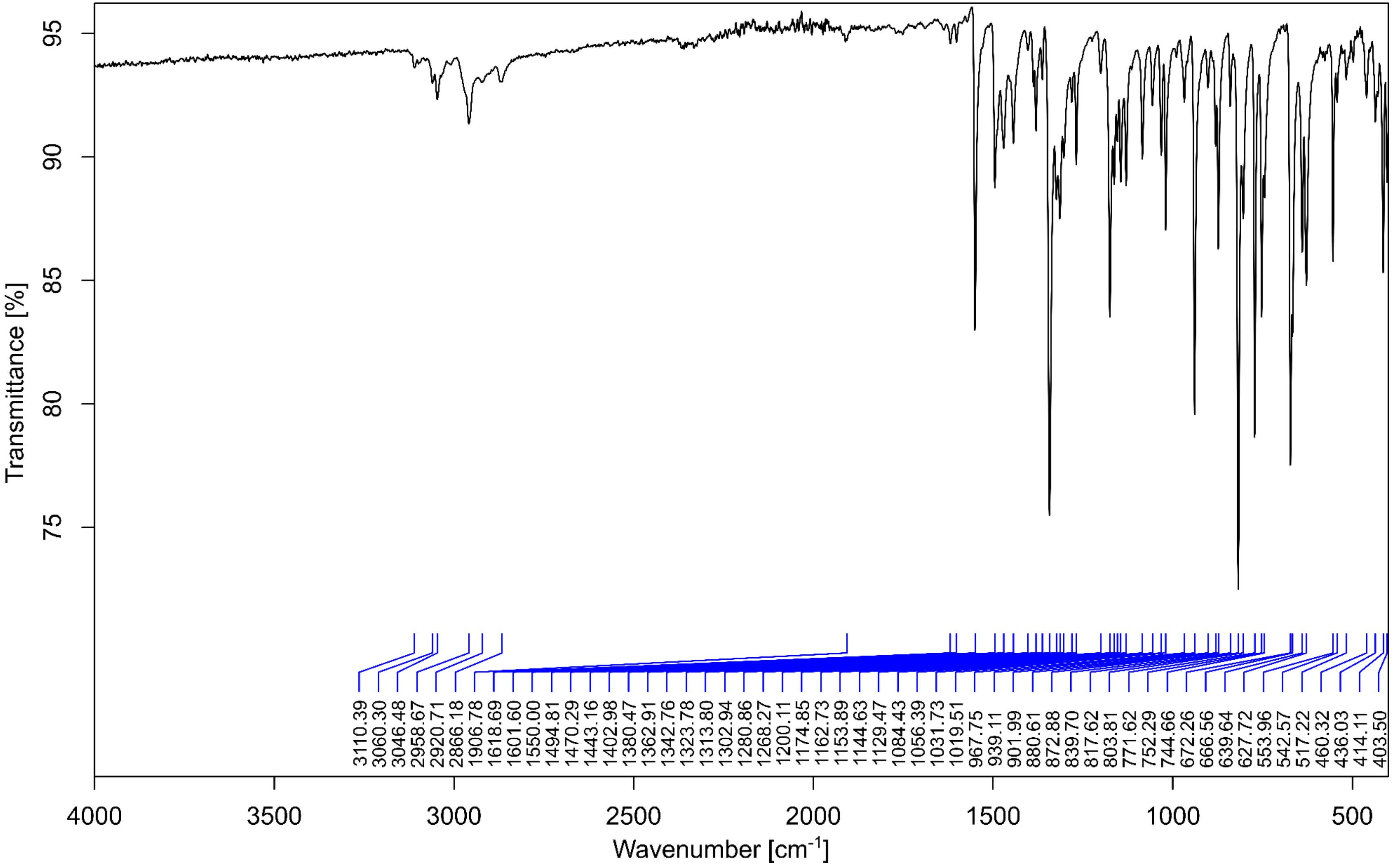

Figure S15: IR spectrum of **1f**.

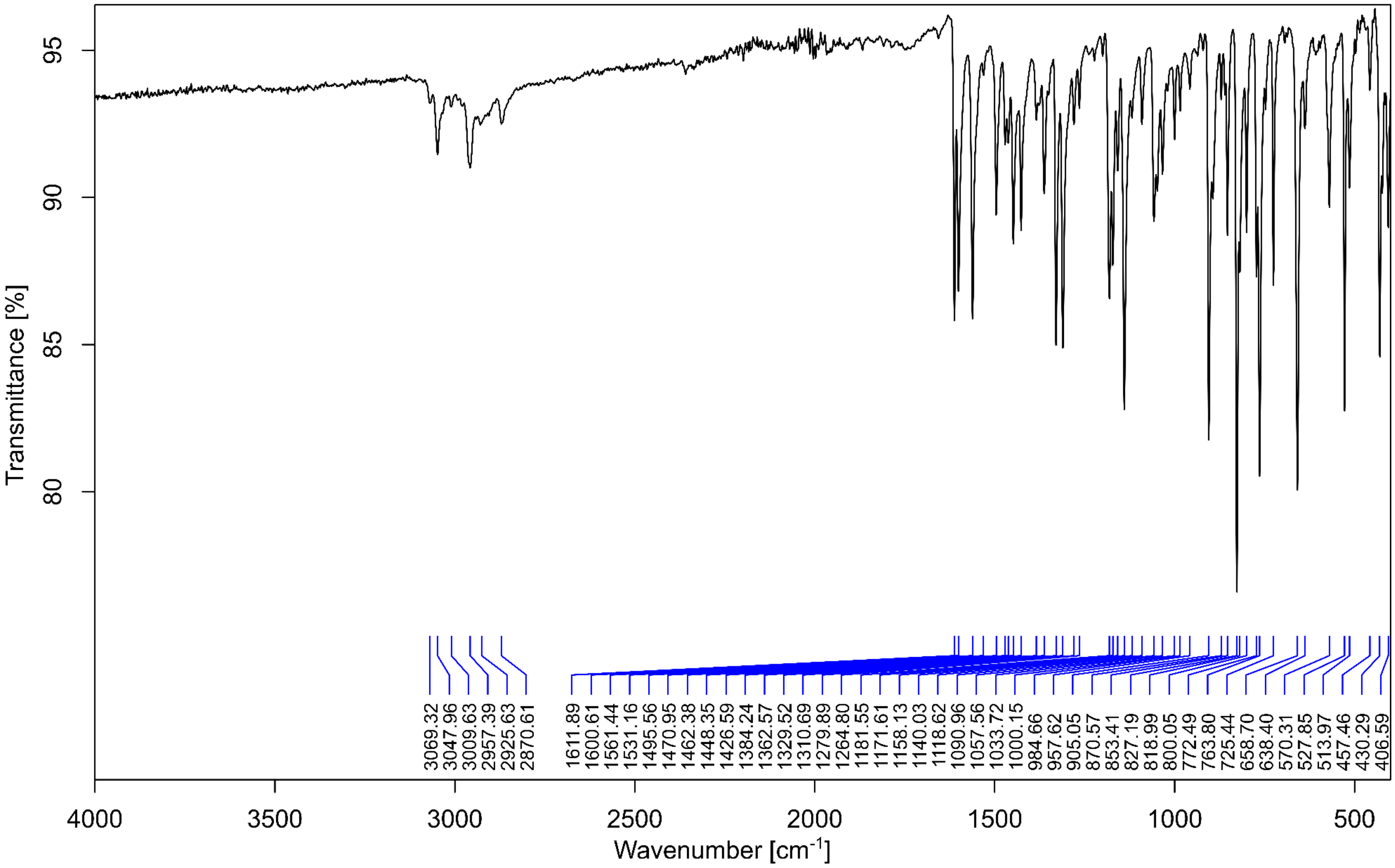

Figure S16: IR spectrum of **1g**.

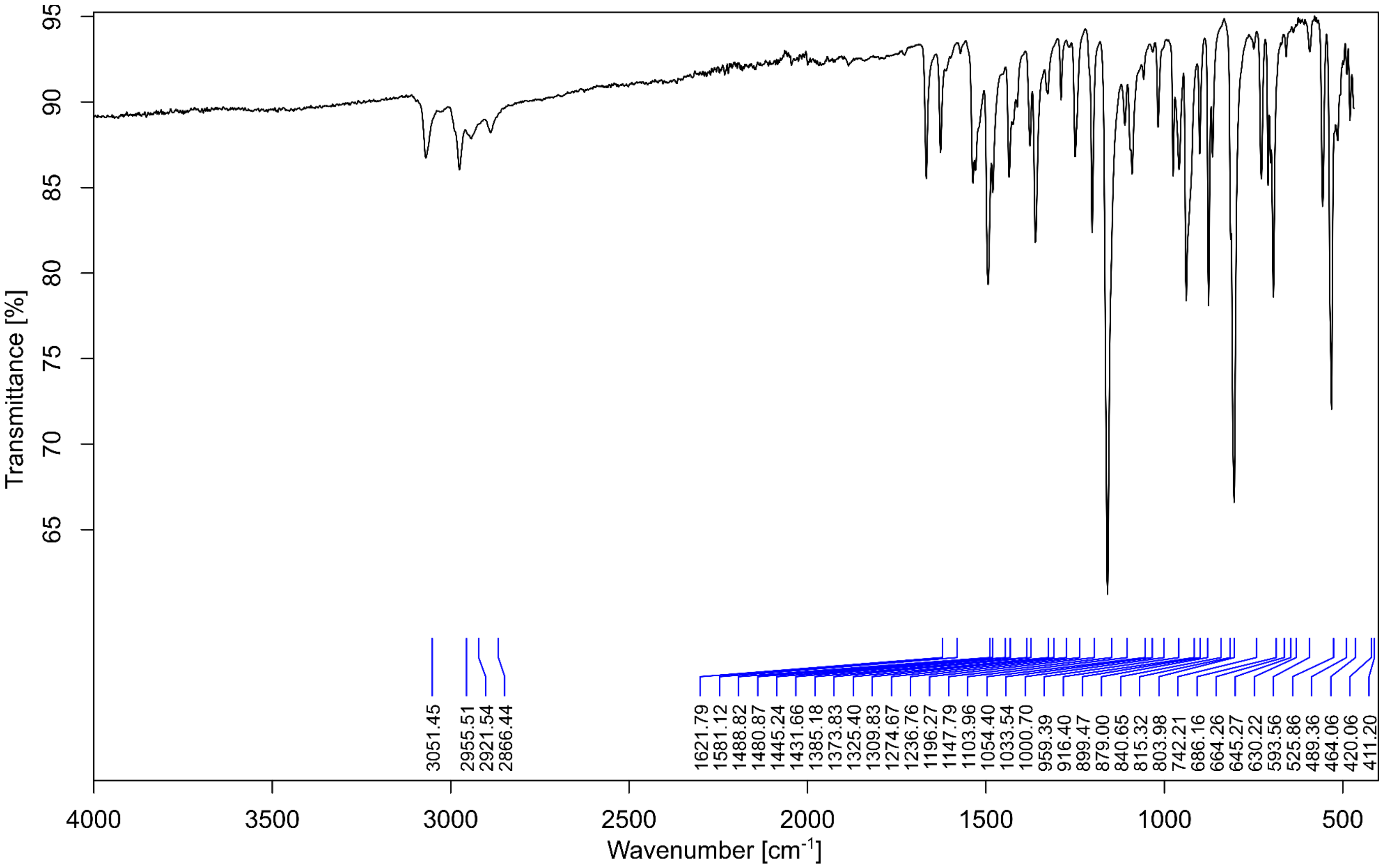

Figure S17: IR spectrum of **1h**.

6. Additional computational data

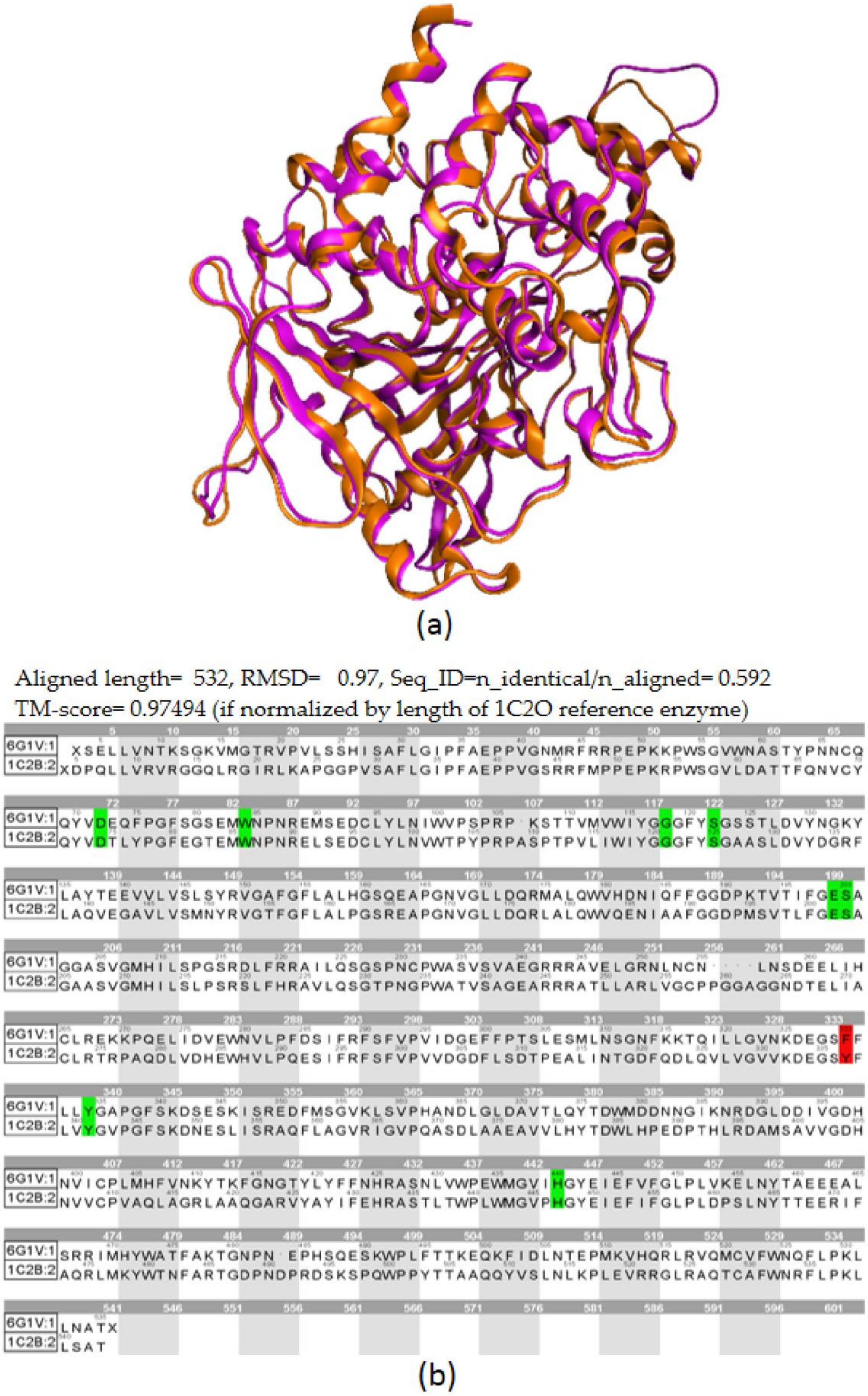

Figure S18. Comparison of the three-dimensional representations of sting ray (*Torpedo californica*) AChE (PDB ID:6G1V; orange) with electric eel AChE (PDB ID: 1C2B; violet) using the TM- alignment, with the obtained result. The identical amino acidic residue in the active sites of both AChEs is marked in green, whereas in red the different amino acidic residues in the active sites are reported.

7. ADME prediction data

1g

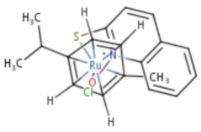

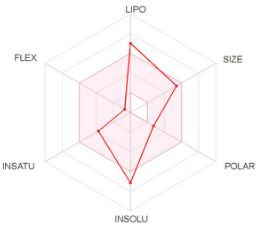

SMILES CC(C12C3[Ru]45672(C1C5C7(C34)C)(Cl)Sc1n(O6)c2ccccc2cc1)C

Physicochemical Properties	
Formula	C19H20ClNORuS
Molecular weight	446.96 g/mol
Num. heavy atoms	24
Num. arom. heavy atoms	10
Fraction Csp3	0.53
Num. rotatable bonds	1
Num. H-bond acceptors	1
Num. H-bond donors	0
Molar Refractivity	96.36
TPSA	39.46 Å^2

Lipophilicity	
Log $P_{o/w}$ (iLOGP)	0.00
Log $P_{o/w}$ (XLOGP3)	6.79
Log $P_{o/w}$ (WLOGP)	6.50
Log $P_{o/w}$ (MLOGP)	5.12
Log $P_{o/w}$ (SILICOS-IT)	2.62
Consensus Log $P_{o/w}$	4.21

Water Solubility	
Log *S* (ESOL)	-7.13
Solubility	3.30e-05 mg/ml ; 7.39e-08 mol/l
Class	Poorly soluble
Log *S* (Ali)	-7.43
Solubility	1.68e-05 mg/ml ; 3.75e-08 mol/l
Class	Poorly soluble
Log *S* (SILICOS-IT)	-3.62
Solubility	1.07e-01 mg/ml ; 2.39e-04 mol/l
Class	Soluble

Pharmacokinetics	
GI absorption	High
BBB permeant	No
P-gp substrate	No
CYP1A2 inhibitor	Yes
CYP2C19 inhibitor	No
CYP2C9 inhibitor	No
CYP2D6 inhibitor	No
CYP3A4 inhibitor	No
Log K_p (skin permeation)	-4.21 cm/s

Druglikeness	
Lipinski	Yes; 1 violation: MLOGP>4.15
Ghose	No; 1 violation: WLOGP>5.6
Veber	Yes
Egan	No; 1 violation: WLOGP>5.88
Muegge	No; 2 violations: XLOGP3>5, #rings>7
Bioavailability Score	0.55

Medicinal Chemistry	
PAINS	0 alert
Brenk	0 alert
Leadlikeness	No; 2 violations: MW>350, XLOGP3>3.5
Synthetic accessibility	5.57

1d

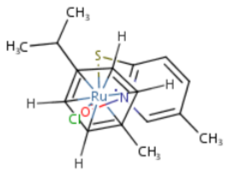

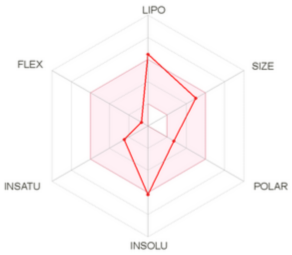

SMILES Cc1ccc2n(c1)O[Ru]13456(S2)(Cl)C2C4C6(C3C1C52C)C(C)C

Physicochemical Properties	
Formula	C16H20ClNORuS
Molecular weight	410.92 g/mol
Num. heavy atoms	21
Num. arom. heavy atoms	6
Fraction Csp3	0.69
Num. rotatable bonds	1
Num. H-bond acceptors	1
Num. H-bond donors	0
Molar Refractivity	83.82
TPSA	39.46 Å^2

Lipophilicity	
Log $P_{o/w}$ (iLOGP)	0.00
Log $P_{o/w}$ (XLOGP3)	5.81
Log $P_{o/w}$ (WLOGP)	5.66
Log $P_{o/w}$ (MLOGP)	4.38
Log $P_{o/w}$ (SILICOS-IT)	2.16
Consensus Log $P_{o/w}$	3.60

Water Solubility	
Log *S* (ESOL)	-6.19
Solubility	2.63e-04 mg/ml ; 6.41e-07 mol/l
Class	Poorly soluble
Log *S* (Ali)	-6.41
Solubility	1.60e-04 mg/ml ; 3.90e-07 mol/l
Class	Poorly soluble
Log *S* (SILICOS-IT)	-3.17
Solubility	2.78e-01 mg/ml ; 6.78e-04 mol/l
Class	Soluble

Pharmacokinetics	
GI absorption	High
BBB permeant	Yes
P-gp substrate	No
CYP1A2 inhibitor	Yes
CYP2C19 inhibitor	No
CYP2C9 inhibitor	No
CYP2D6 inhibitor	No
CYP3A4 inhibitor	No
Log K_p (skin permeation)	-4.68 cm/s

Druglikeness	
Lipinski	Yes; 1 violation: MLOGP>4.15
Ghose	No; 1 violation: WLOGP>5.6
Veber	Yes
Egan	Yes
Muegge	No; 2 violations: XLOGP3>5, #rings>7
Bioavailability Score	0.55

Medicinal Chemistry	
PAINS	0 alert
Brenk	0 alert
Leadlikeness	No; 2 violations: MW>350, XLOGP3>3.5
Synthetic accessibility	5.46

Figure S19. ADME Prediction of compounds **1g** and **1d** evaluated by online Server Swiss-ADME.
